# MXene-polydopamine-cyanobacteria photobioelectrochemical biosensor for atrazine detection in environmental waters

**DOI:** 10.1007/s00604-026-08471-z

**Published:** 2026-10-23

**Authors:** Caio César Gonçalves Silva, Maiza de Lima Bueno, Max Fabricio Falone, Lucas Felipe de Lima, William Reis de Araujo, Maria Valnice Boldrin Zanoni, Maisa Azevedo Beluomini

**Affiliations:** 1 Faculdade de Ciências Agrárias e Veterinárias, Universidade Estadual Paulista (UNESP), Jaboticabal, São Paulo Brasil; 2 Instituto de Química, Universidade Estadual Paulista (UNESP), Araraquara, São Paulo Brasil; 3 Departamento de Química Analítica, Instituto de Química, Universidade Estadual de Campinas (UNICAMP), Campinas, São Paulo Brasil

**Keywords:** Photosynthesis, Cyanobacteria, MXene, Polydopamine, Atrazine, Herbicide

## Abstract

A cyanobacteria-based photobioelectrochemical biosensor was developed for the first time using *Synechococcus elongatus* whole cells immobilized on a Ti₃C₂Tₓ MXene electrode modified with an electropolymerized polydopamine (PDA) layer. This surface created a biotic–abiotic interface that facilitated light-driven electron transfer for the sensitive screening of photosystem II (PSII)-inhibiting herbicides in environmental waters, using atrazine (ATZ) as a model analyte. The biosensor was characterized by scanning electron microscopy (SEM), electrochemical impedance spectroscopy (EIS), X-ray photoelectron spectroscopy (XPS), and Fourier-transform infrared spectroscopy (FTIR). The detection strategy relies on the inhibition of PSII photosynthetic activity, which produces a concentration-dependent decrease in photocurrent. Under optimized conditions, the biosensor achieved a limit of detection (LOD) of 1.92 nM (0.414 ppb), a linear range of 5–200 nM, and a sensitivity of 177.71 nA decade⁻¹ cm⁻². Interference studies demonstrated class selectivity toward PSII-inhibiting herbicides (e.g., DCMU, simazine, desethylatrazine), with negligible response (< 10%) from non-PSII pesticides and common inorganic ions. Regarding operational stability, the device retained > 95% for 1 day and approximately 93% at day 2. However, a 61% signal loss was observed by day 5 (stored at 4 °C), highlighting an operational limitation inherent to whole-cell biological systems. The biosensor was successfully applied to determine ATZ in groundwater, river water, and seawater, delivering accurate recoveries (99.75–103.9%) and offering a green alternative for on-site environmental monitoring.

**Graphical abstract:**

**Figure:**
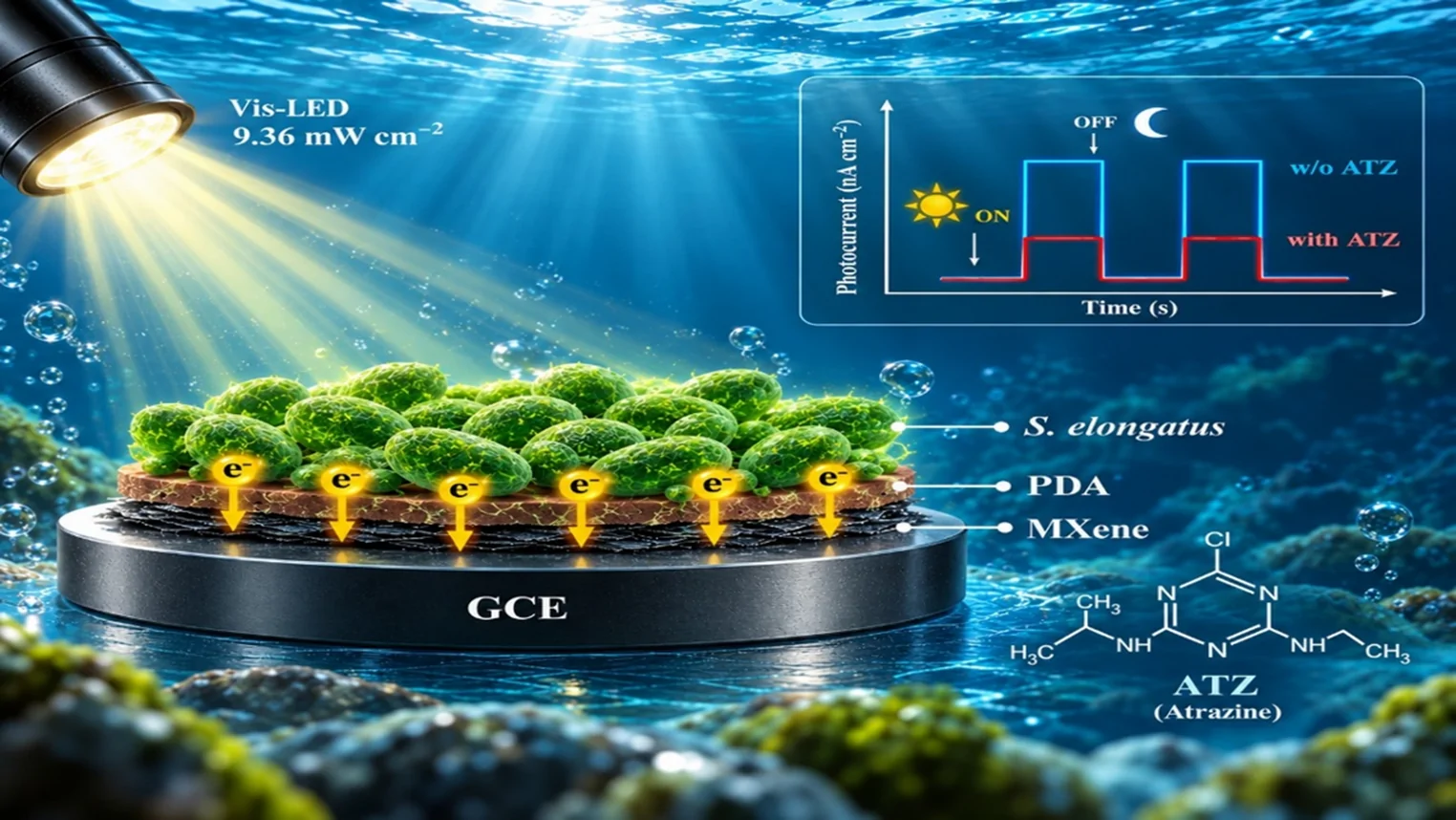

**Supplementary Information:**

The online version contains supplementary material available at https://doi.org/10.1007/s00604-026-08471-z.

## Introduction

Atrazine (ATZ), 1-chloro-3-ethylamino-5-isopropylamino-2,4,6-triazine, is one of the most frequently detected triazine herbicides in environmental [1] and food samples [2, 3]. Its intensive use in important crops such as maize, sugarcane, and sorghum, combined with high chemical stability and slow degradation, results in pronounced environmental persistence [4]. Consequently, chronic ATZ exposure has been linked to ecological [5] imbalance and adverse effects on non-target organisms [6], raising continued concerns regarding its potential risks to environmental and human health. In response to these concerns, regulatory agencies have established maximum allowable concentrations for pesticides in water. For example, the U.S. Environmental Protection Agency (EPA) sets a maximum concentration of 3.0 µg L⁻¹ in drinking water for ATZ [7]. This regulatory threshold highlights the need for sensitive, reliable, and accessible analytical strategies capable of detecting ATZ at environmentally relevant concentrations.

The main approaches to detect triazine herbicides are based on chromatographic techniques (e.g., liquid chromatography (HPLC) or gas chromatography (GC), which have been the backbone of pesticide monitoring for decades [8]. These methods combine high sensitivity and selectivity with robust quantification. However, their drawbacks are well known, because they frequently require laborious and time-consuming sample preparation (e.g., extraction, cleanup, concentration steps), depend on expensive, non-portable instrumentation, and are often unsuitable for on-site or real-time monitoring, limiting their practicality for extensive environmental surveillance or rapid food-safety screening. Therefore, the development of new analytical approaches to economical, sensitive, and fast devices for on-site environmental herbicide monitoring would be of great relevance.

Photobioelectrochemical sensors represent a successful emerging class of analytical devices that integrate photosynthetic units (e.g., isolated photosystems [9], thylakoid membrane [10], chloroplast [11], microalgae [12], or purple bacteria [13]) immobilized on an electrode surface [14]. By converting light-driven biochemical processes into measurable electrical signals, these platforms enable sensitive and rapid detection of triazine herbicides that interfere with photosynthetic activity. While isolated components such as purified photosystems or subunits can offer better electron transfer pathways [15], their practical application is often limited by complex preparation procedures, reduced long-term stability, and susceptibility to environmental conditions. In contrast, whole-cell systems preserve the native cellular architecture, enabling self-repair mechanisms, metabolic regulation, and sustained photosynthetic activity [16, 17], thereby representing an attractive alternative for use in biosensor devices.

Among photosynthetic microorganisms, cyanobacteria have attracted attention in photobioelectrochemical platforms [18], although their application in such biosensor devices remains relatively underexplored. These organisms are easy to cultivate, grow rapidly, and respond sensitively to a wide range of environmental conditions, making them suitable for environmental monitoring applications. Importantly, cyanobacteria perform oxygenic photosynthesis [19] using a photosynthetic apparatus that is structurally and functionally analogous to that of higher plants, with photosystem II (PSII) playing a central role in light-driven electron transfer by water oxidation. PSII is a well-established molecular target of photosynthesis-inhibiting herbicides [20, 21], particularly triazines such as ATZ, so these compounds competitively bind to the secondary quinone electron acceptor (Q𝐵) site of the D1 protein, thereby blocking electron transport from PSII and suppressing photosynthetic activity.

Despite the advantages of whole-cell photobioelectrochemical systems, the extraction of photogenerated electrons from the photosynthetic chain remains a challenge. In cyanobacterial cells, the complexity of biological membrane structure constitutes an effective barrier between the intracellular electron-transfer chain and the electrode surface, resulting in inefficient direct electron transfer [22]. To overcome this limitation, strategies involving exogenous diffusible redox mediators and polymer-based mediators emerge as promising alternatives.

Redox polymers, particularly polydopamine (PDA), have been successfully employed as electron mediators for the immobilization and electrical wiring of photosynthetic systems, including chloroplasts [23], microalgae [14], and intact bacterial cells [24]. PDA-based matrices incorporate redox-active functional groups, such as quinones and semiquinones, which facilitate efficient electron transfer between biological components and electrode surfaces [25]. Beyond its mediating capability, PDA exhibits many advantageous properties, including strong adhesion to a wide range of substrates, low cytotoxicity, photoactivity arising from light absorption in the visible region, and a simple and versatile synthesis process [26].

In addition to efficient biological–electrode surface communication, the performance of photobioelectrochemical devices is influenced by the physicochemical properties of the electrode interface. The incorporation of nanomaterials (e.g., carbon-based and metallic nanoparticles) has emerged as an effective strategy to enhance electron transfer kinetics, increase active surface area, and improve cell–electrode interactions in whole-cell photobiohybrid devices [27, 28]. Among these nanomaterials, MXenes, particularly Ti₃C₂Tₓ, have attracted growing attention due to their electrical conductivity, hydrophilicity, biocompatibility, and high surface area [29, 30]. These features enhance the electrode surface for biological immobilization and electron transport, making MXenes an attractive material for photobioelectrochemical sensing platforms.

In this study, we report for the first time the development of an innovative photobioelectrochemical sensor based on whole cyanobacterial cells immobilized on an MXene-modified electrode integrated with a PDA redox matrix layer for the detection of ATZ in water samples. The proposed photobiohybrid platform was systematically evaluated in terms of photocurrent generation and analytical performance, including sensitivity, linear range, limit of detection, and limit of quantification.

## Experimental

### Reagents and solutions

All reagents employed in this study were of analytical or research grade (~ 98%). The aqueous solutions were prepared using ultrapure water (Milli-Q SQ2 Series, Merck) with a resistivity of 18.2 MΩ·cm at 25 °C. Potassium ferricyanide (K₃[Fe(CN)₆]), potassium ferrocyanide (K₄[Fe(CN)₆]), potassium chloride (KCl), potassium dihydrogen phosphate (KH₂PO₄), dipotassium hydrogen phosphate (K₂HPO₄), lithium fluoride (LiF), titanium–aluminum carbide (Ti₃AlC₂), hydrochloric acid, and dopamine were purchased from Sigma-Aldrich.

### MXene synthesis and characterization

Detailed information regarding the synthesis, physicochemical, and electrochemical characterization of MXene is available in the Supporting Information. The corresponding results and discussions include FEG-SEM and FTIR analyses (Fig. S2), XPS analysis (Fig. S3), and cyclic voltammetry (CV) characterization (Fig. S4).

### Cyanobacteria growth conditions

The cyanobacteria *Synechococcus elongatus* was acquired from the Aida and Kutner Microorganism Bank (BMAK) at the Oceanographic Institute of the University of São Paulo (USP) and was registered in the Brazilian National System for the Management of Genetic Heritage and Associated Traditional Knowledge (SisGen) under registration number A997066. Cultures were grown in 50 mL of Blue Green-11 (BG-11) medium in 125 mL Erlenmeyer flasks at (23 ± 2) °C, with orbital shaking at 160 rpm, and illuminated by a visible LED (Vis-LED) system under a 12 h light:12 h dark photoperiod. The exponential growth phase occurred between days 2 and 8 (Fig. S1). The biomass was used to fabricate the biosensor after 48 h of cultivation.

### Photoelectrochemical biosensor fabrication and setup

**Fig. 1:**
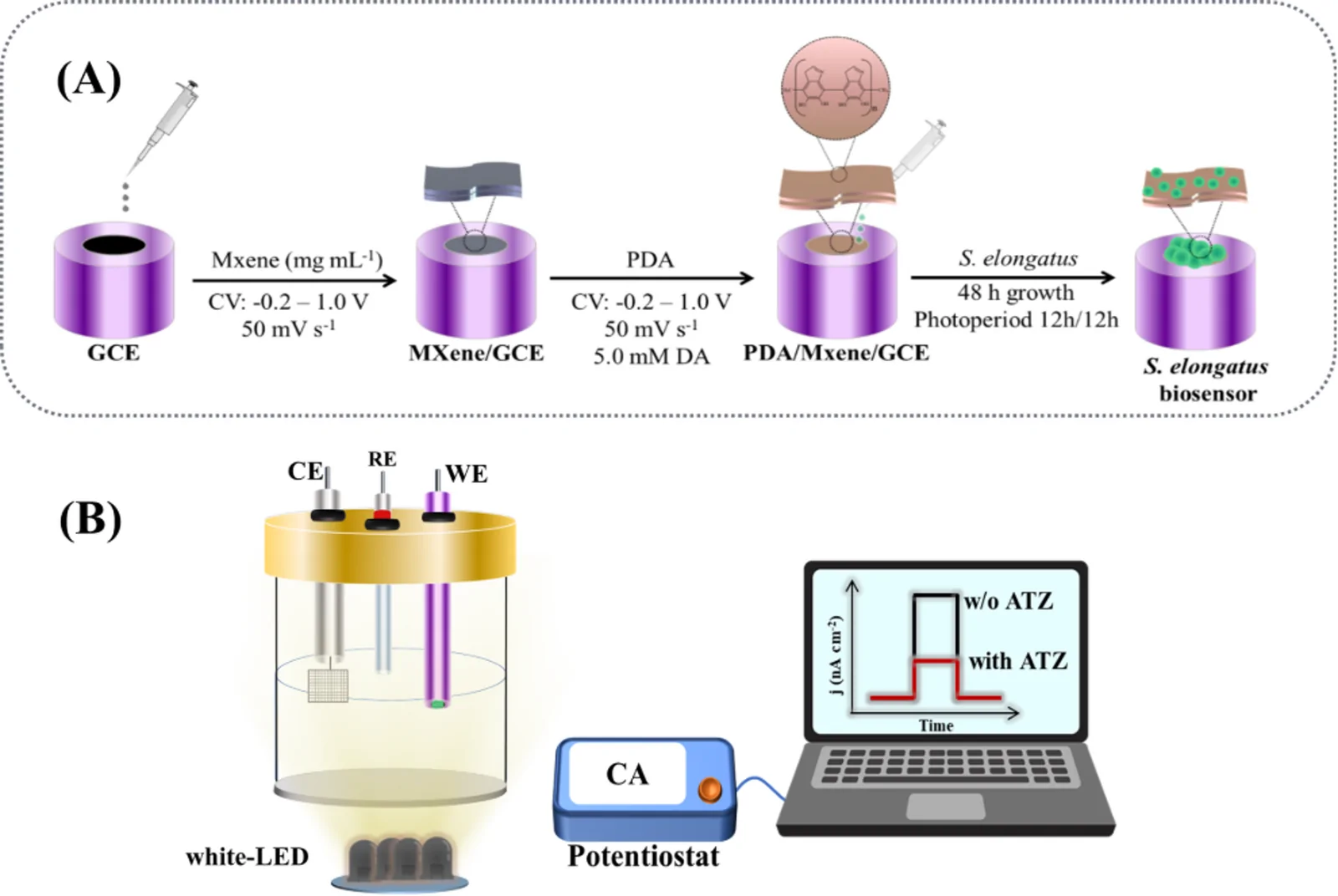
Schematic illustration of the photoelectrochemical biosensor. (**A**) Step-by-step representation of the biosensor fabrication process, including electrode surface modification and cyanobacteria biofunctionalization. (**B**) Scheme of the photobioelectrochemical workstation used for measurements, comprising the photoelectrochemical cell, light source, and current acquisition system

Biosensor fabrication was conducted using a conventional three-electrode electrochemical cell (Fig. 1), comprising an Ag/AgCl (3.0 mol L⁻¹ KCl) reference electrode, a platinum wire counter electrode, and a glassy carbon working electrode, GCE (3.0 mm diameter, geometric area 0.0707 cm²). The stepwise electrode modification protocol is depicted in Fig. 1A: initially, 5 µL of a 2.0 mg mL⁻¹ MXene dispersion for initial studies was drop-cast onto the polished WE and dried at room temperature to yield MXene/GCE, and the MXene concentration condition was subsequently optimized to 0.5 mg mL^− 1^. The MXene-modified electrode was then functionalized with a polydopamine (PDA) layer through electropolymerization of 5.0 mM dopamine in 100 mM PBS (pH 7.0) by cyclic voltammetry (CV), in a potential range of − 0.2 V to + 1.0 V (vs. Ag/AgCl, 3 M KCl) at a 50 mV s^−¹^ for multiple cycles [31]. The cyanobacteria cells, cultured for 48 h, were harvested by centrifugation at 4000 rpm for 10 min and washed three times with 100 mM PBS (pH 7.0) to remove residual culture medium, and resuspended to OD_680nm_ = 5.0. A 5 µL aliquot of this suspension was then drop-cast onto the PDA/MXene/GCE and dried at room temperature. To prevent cell detachment during photoelectrochemical measurements, the immobilized cells were covered with a dialysis cellulose membrane (molecular weight cutoff 14 kDa, Sigma-Aldrich). Figure 1B illustrates the complete photobioelectrochemical setup, featuring a conventional three-electrode cell interfaced with a PalmSens4 electrochemical workstation (PalmSens BV) coupled to a microcomputer via PSTrace software for data acquisition. A homemade white-LED system (maximum power density: 9.36 mW cm⁻²) served as the light source to stimulate cyanobacterial photosynthetic activity, with performance evaluated by chronoamperometry (CA) under chopped-light illumination at 23 ± 2 °C. All measurements were conducted in 0.1 M PBS (pH 7.0), monitoring the photocurrent response in the presence (w/ atrazine) and absence (w/o atrazine) of the target analyte.

### Electroanalytical measurements

The biosensor was immersed in 100 mM PBS (pH 7.0), and chronoamperometric measurements were performed at an applied potential of + 0.6 V vs. Ag/AgCl. For addition of ATZ, the photocurrent was first allowed to stabilize before analyte injections. The analytical performance was evaluated by constructing a calibration curve, from which figures of merit such as linear concentration range, limit of detection (LOD), and limit of quantification (LOQ) were calculated according to IUPAC guidelines using the equations LOD = 3.3 *SD*/m and LOQ = 10 *SD/m*, where *SD* represents the standard deviation of the regression in relation to the calibration curve, and *m* is the slope and represents the sensitivity of the device [32], and the antilog was taken. To verify accuracy in real matrices, river water, seawater, and groundwater samples were collected in São Paulo State. These samples were filtered through a 0.5 μm paper filter to remove all particulate matter and kept in a refrigerator until further use. After this, a 100 mM PBS (pH 7.0) was prepared using these aqueous samples, which were spiked with 80 nM ATZ. The recovery percentages were determined by the analytical curve interpolation method, based on the ratio between measured and nominal spiked concentrations. All experiments were performed in triplicate (*n* = 3). So, the evaluation of the proposed procedure was realized using 100% of the samples in preparation of the supporting electrolyte, without any clean-up or pre-treatment steps, except for the preliminary filtration to remove particulate matter in the samples.

### Chromatographic analysis: LC-MS/MS

To evaluate the recovery of ATZ, liquid chromatography coupled with tandem mass spectrometry (LC–MS/MS) was applied. Samples were spiked with 80.0 nM ATZ and processed following the corresponding analytical protocol. The recovered concentrations were determined by interpolation using external calibration curves. Before analysis, all samples were filtered through 0.22 μm nylon syringe membranes. Chromatographic analyses were carried out on a Shimadzu Nexera X2 UHPLC system equipped with an LC-30 CE binary pump, SIL-30 AC autosampler, and CTO-20 A column oven. Separation was performed on a Phenomenex Kinetex C18 analytical column (150 × 3.0 mm, 5 μm particle size, 100 Å pore size) maintained at 40 °C.

The mobile phase consisted of water containing 0.1% (v/v) formic acid (solvent A) and acetonitrile (solvent B), delivered at a flow rate of 1.0 mL min⁻¹. The gradient program was as follows: 30% B from 0 to 2 min, a linear increase to 50% B from 2 to 15 min, a return to 30% B between 15 and 15.5 min, and re-equilibration at 30% B until 21 min. The injection volume was 30 µL. To protect the mass spectrometer from non-relevant eluents, the column effluent was diverted to waste outside the 2.5–17 min acquisition window.

Detection was performed using an AB SCIEX 3200 QTRAP triple quadrupole/linear ion trap mass spectrometer operating with positive electrospray ionization (ESI+). The ion source was operated with an IonSpray voltage of 5500 V and a source temperature of 650 °C. Curtain gas was maintained at 10 psi, while ion source gases 1 and 2 were set at 55 and 55 psi, respectively. Nitrogen was used as the collision gas under medium CAD settings. Quantification was achieved in multiple reaction monitoring (MRM) mode using the transitions m/z: 216.1 → 174.1 (collision energy = 19 eV), 216.1 → 104.2 (collision energy = 37 eV), 216.1 → 96.2 (collision energy = 33 eV), and 216.1 → 68.1 (collision energy = 47 eV), which were employed as qualifier ions for confirmation.

### Biosensor characterizations

The morphological and structural properties of the biophotoelectrode were investigated using scanning electron microscopes with field emission gun (SEM-FEG) of JOEL, models JSM-7500-F and JSM-7401-F. FTIR characterization was performed using an Agilent Cary 630 FTIR spectrometer in transmission mode over a wavenumber range of 400–4000 cm⁻¹. Samples were prepared as KBr pellets, and the spectra were acquired using 128 scans per spectrum. Electrochemical impedance spectroscopy (EIS) measurements were performed using a potential of 0.22 V, frequency range from 0.01 Hz to 100 kHz, with 10 points/decade, potential amplitude of 10 mV, and 5.0 mM [Fe(CN)_6_]^3−^ in 0.1 M KCl solution as the electrochemical probe.

## Results and discussion

### Morphological and electrochemical characterization of the cyanobacteria-based biosensor

**Fig. 2:**
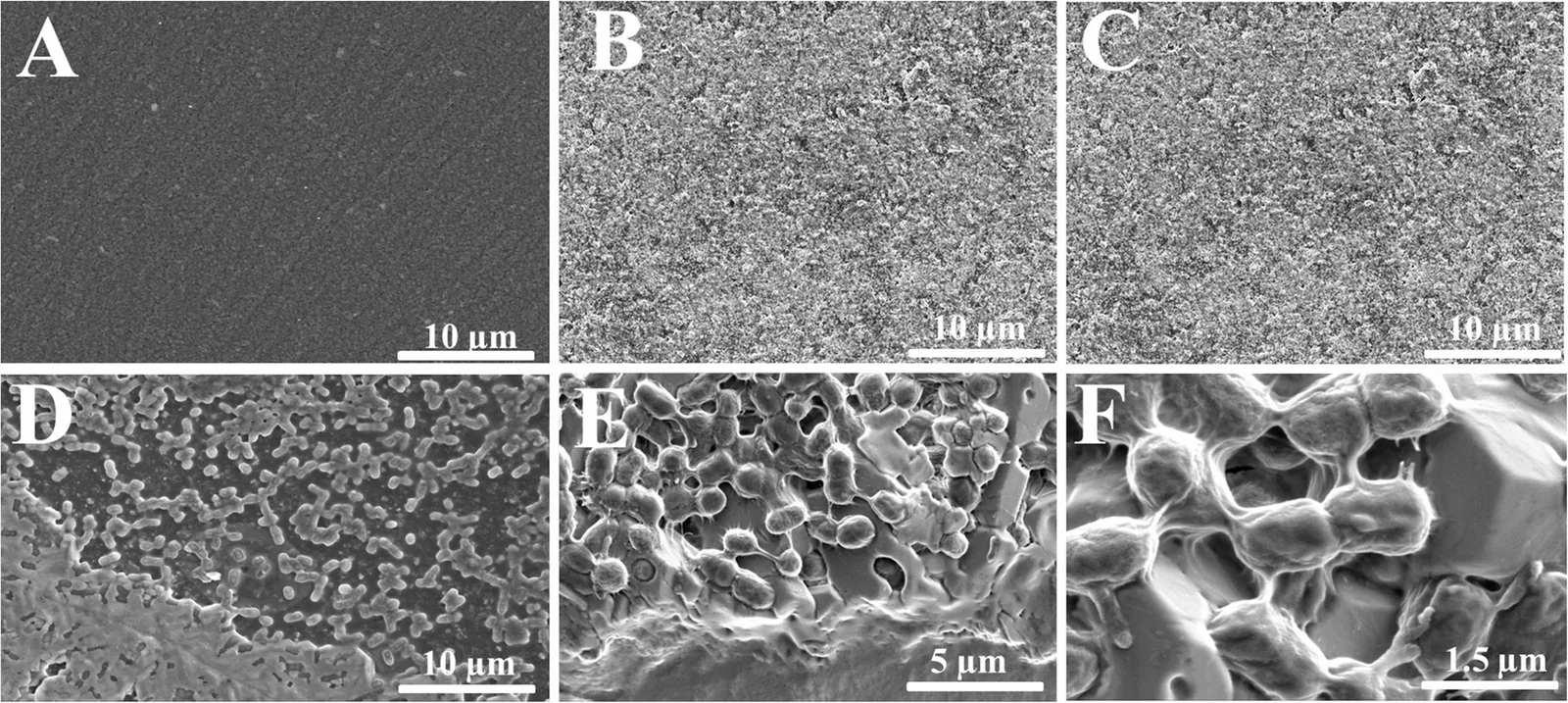
FEG-SEM images of (**A**) GCE, (**B**) MXene/GCE, (**C**) PDA/MXene/GCE, and (**D**, **F**) S. elongatus/PDA/MXene/GCE. Images (**A**, **D**) were acquired at the same magnification (2,500×), whereas images (**E**) and (**F**) were acquired at 10,000× and 25,000×, respectively

SEM analysis was employed to verify the successful stepwise modification of the GCE during cyanobacteria-based biosensor fabrication. Figure 2 presents top-view SEM micrographs showing the progressive surface modifications. Figure 2A depicts the pristine, unmodified GCE, followed by Fig. 2B, which reveals a uniform MXene coating on the GCE surface, characterized by crumpled morphologies featuring numerous ridges and rough topography. Figure 2C shows the surface of the MXene/GCE after PDA coating conforms to the irregular morphology, preserving a heterogeneous and porous surface without evidence of a compact layer that completely masks the underlying structural features. It is important to mention that surface roughness plays a crucial role in cell adhesion and the development of robust biofilms [12, 24]. Following immobilization of *S. elongatus* (Fig. 2D), the presence of rod-shaped cyanobacterial cells homogeneously distributed across the surface confirmed successful immobilization. The average size of *S. elongatus* cells immobilized on the PDA/MXene/GCE surface was 1.60 μm, as shown in Fig. 2E.

EIS was employed to characterize the modified electrode using a 5.0 mM [Fe(CN)₆]³⁻^/^⁴⁻ solution in 0.1 mol L⁻¹ KCl as the redox probe. The typical Nyquist plot exhibits a well-defined semicircle at high frequencies, followed by a linear region at low frequencies, as shown in Fig. S5. An equivalent circuit corresponding to the Nyquist diagram is shown in the inset. In this equivalent circuit, $\:{R}_{s}$ stands for the resistance of the electrolyte solution, the CPE represents a constant phase element associated with the double-layer capacitance, and *R*_*ct*_ corresponds to the charge transfer resistance [33]. The data were analyzed in terms of *R*_*ct*_ due to its straightforward physical meaning compared to other elements and its ability to describe the rate of charge transfer during the electrode reaction. The *R*_*ct*_ value for the bare GCE was 0.34 kΩ while the MXene electrode showed a much lower *R*_*ct*_ of 0.21 kΩ, reflecting its high intrinsic conductivity and efficient interfacial electron transfer. After PDA deposition, *R*_*ct*_ increased to 0.78 kΩ due to the less conductive property of PDA compared to MXene. Interestingly, the incorporation of cyanobacterial cells further increased*R*_*ct*_ to 4.97 kΩ, likely reflecting the insulating nature of the biological membrane layer [23].

 Despite the increased resistance, the biophotoanode photoelectrochemical performance was improved, as the biological interface enabled light-driven charge separation and redox mediation. This behavior is also supported by the calculated electron transfer rate constant (k^0^) using a Butler-Volmer equation [34], which decreased progressively from 3.59 × 10⁻⁶ cm·s⁻¹ (MXene) to 9.66 × 10⁻⁷ cm·s⁻¹ (PDA/MXene) and 1.51 × 10⁻⁷ cm·s⁻¹ (*S.elongatus*/PDA/MXene/GCE), as summarized in Table S1. These values are consistent with the expected kinetic limitations introduced by the PDA and external membrane of cyanobacteria, but do not preclude effective electron transfer under illumination conditions.

### Performance of cyanobacteria-based biosensor

**Fig. 3:**
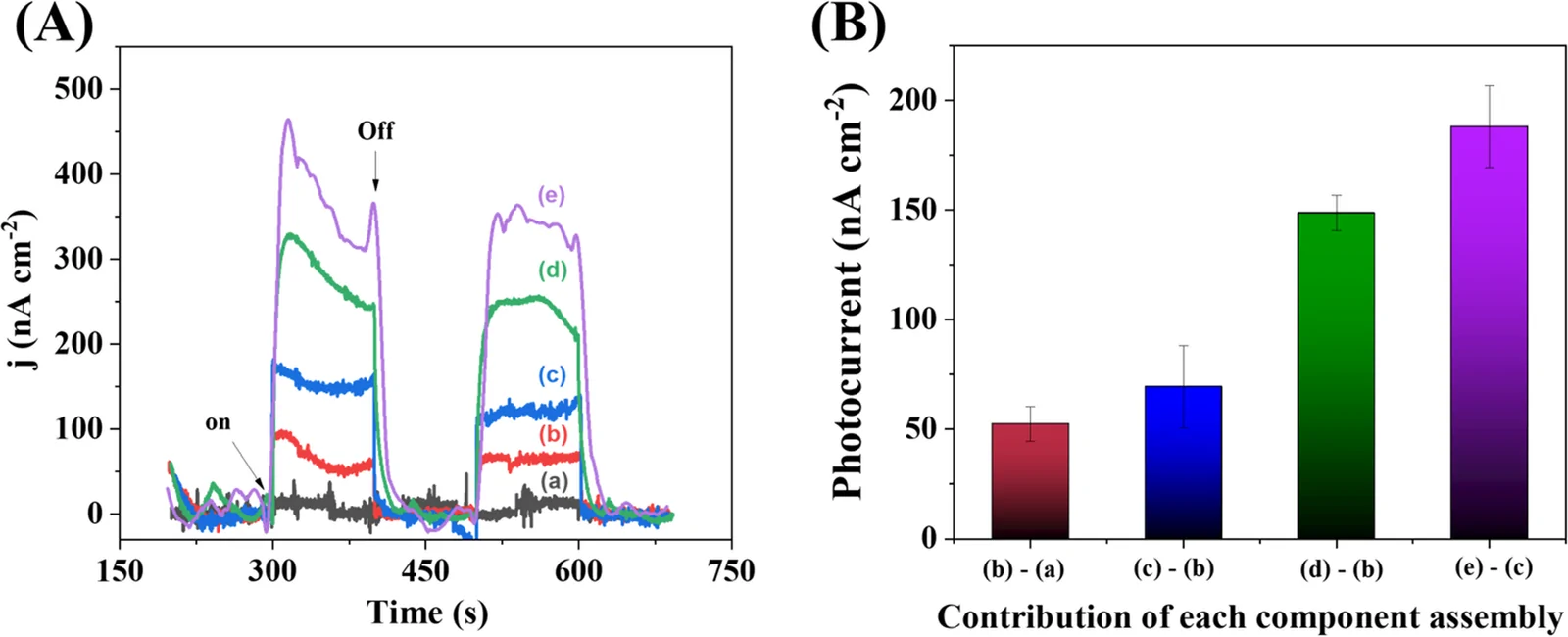
(**A**) Chronoamperometric responses under chopped Vis-LED illumination (9.36 mW cm^-2^) for photobiohybrid electrode with: (**a**) MXene, (**b**) PDA (10 cycles), (**c**) PDA - *S. elongatus*, (**d**) MXene - PDA, and (**e**) MXene- PDA- *S. elongatus*. (**B**) Comparative analysis of the photocurrent responses of the systems shown in panel A, evidencing the contributions of each component. Electrolyte: 100 mM PBS, pH 7.0; Eapp: +0.6 V vs. Ag/AgCl/3.0 M KCl,*S. elongatus* (OD₆₈₀ = 5.0), [Mxene]= 2.0 mg mL^-1^, and 10 cycles of electropolymerization of PDA. Error bars represent standard deviation (*n* = 3 different electrodes)

The current density responses in the different components of the photobioelectrochemical sensor were investigated by chopped-light chronoamperometry, as shown in Fig. 3A. The electrode modified with MXene (curve a) exhibited a low photocurrent density of 20.96 nA cm⁻² under visible light. This response is consistent with the optical properties of MXenes, which exhibit negligible absorption in the visible region of the electromagnetic spectrum, thereby limiting direct photoinduced charge carrier generation [35, 36].

To improve the photoelectrochemical performance of the biosensor, a PDA layer (curve b) was deposited onto the electrode surface to act as a photoactive material and a matrix for cyanobacterial immobilization. Upon modification, the photocurrent density increased to 73.26 nA cm⁻². This enhancement was attributed to PDA absorption in the visible region, which promotes efficient charge separation through its conjugated catechol/quinone structure, thereby reducing electron–hole recombination and facilitating interfacial electron transfer [37, 38]. Following PDA deposition, *S. elongatus* cells were immobilized on the electrode surface (curve c), resulting in a further increase in photocurrent density to 142.58 nA cm⁻². This behavior demonstrates efficient interaction between the cyanobacterial photosynthetic apparatus and the electrode surface, leading to electron generation under visible-light illumination. A fraction of these photoexcited electrons is transferred from the cells to the electrode through the PDA layer, confirming the establishment of photobioelectrochemical communication between the cell and inorganic components. This observation is in agreement with previous reports describing photocurrent production [14, 39].

When MXene and PDA were combined before biological functionalization (curve d), a photocurrent density of 222.25 nA cm⁻² was obtained, surpassing the responses observed for either material alone. This enhancement indicates an interaction between MXene and PDA, wherein the high conductivity, large surface area, and metallic-like behavior of MXene not only facilitate rapid electron transport and collection but also provide abundant active sites for PDA anchoring. Meanwhile, PDA contributes visible-light absorption, interfacial charge mediation, and surface stabilization. Together, these complementary properties lead to improved interfacial electron transfer efficiency and create a biofriendly surface for subsequent cell immobilization.

The highest photocurrent density was achieved with the fully assembled biohybrid system (curve e), reaching 330.48 nA cm⁻² under visible light illumination. This substantial enhancement highlights the effective integration of light harvesting, biological electron generation, redox mediation, and conductive charge transport within a single architecture. In this configuration, the PDA acts as a multifunctional interface, simultaneously promoting strong cell adhesion, facilitating electron transfer from the photosynthetic apparatus to the electrode, and efficiently coupling to the large-surface-area, conductive MXene network.

Figure 3B compares the photocurrent densities at each stage of photobioelectrode construction, calculated from the average of two on/off cycles. The results highlight the contributions of MXene, PDA, and *S. elongatus* to the photoelectrochemical response, confirming the importance of the biohybrid interface for photobioelectrode performance.

### Biophotoanode optimizations

**Fig. 4:**
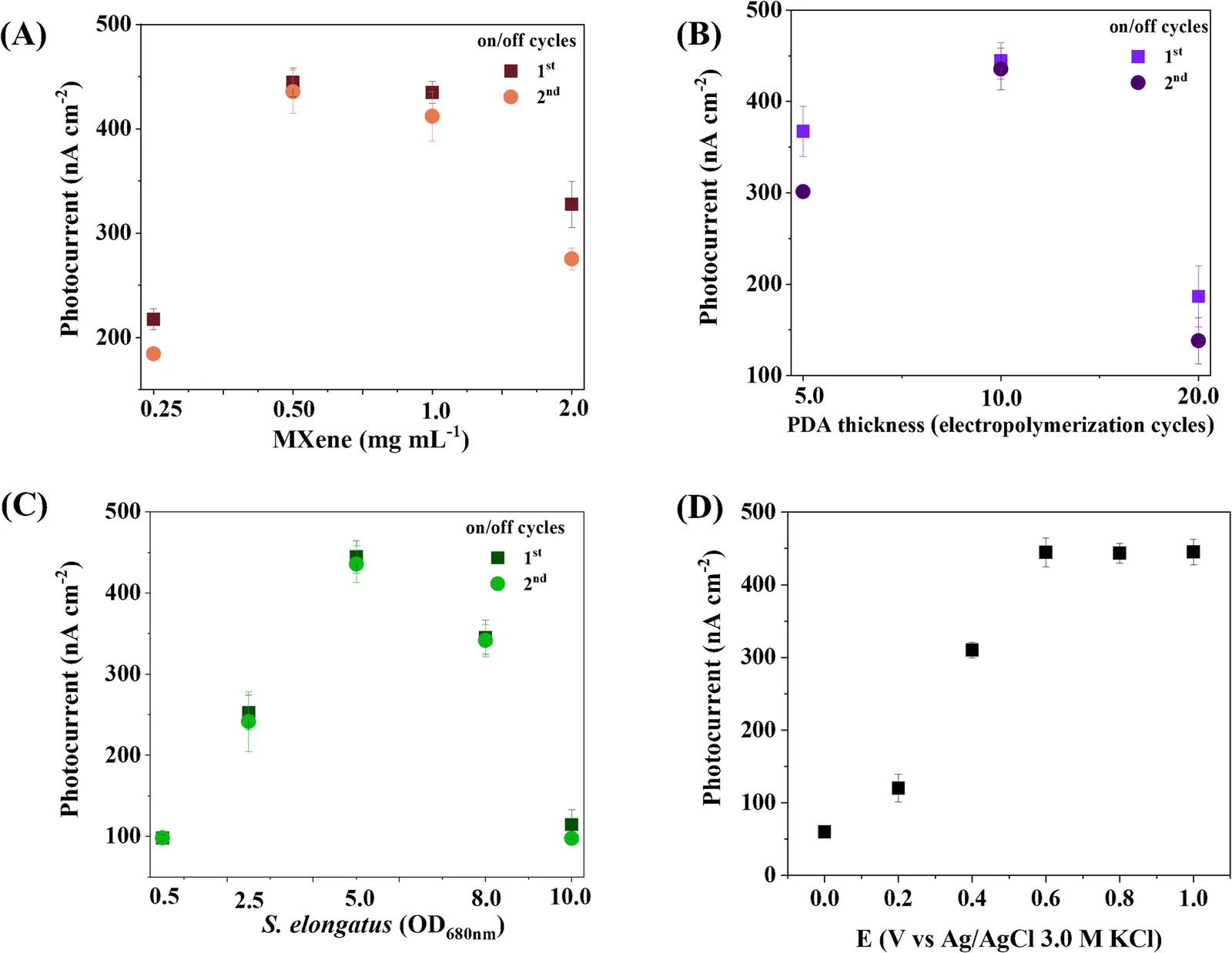
Effect of (**A**) different amounts of MXene, (**B**) PDA thickness layer, (**C**) the OD680 nm of *S. elongatus*, (**D**) applied voltage in the photobiohybrid platform. Electrolyte: 100 mM PBS, pH 7.0; Eapp: 0.6 V vs. Ag/AgCl/3.0 M KCl. Error bars represent standard deviation (*n* = 3 different electrodes)

To achieve optimal biosensor performance, key parameters were systematically optimized, including MXene concentration, PDA layer thickness, optical density of immobilized *S. elongatus* cells on the electrode surface, and applied potential. The first parameter optimized was the MXene concentration deposited on the GCE surface. As shown in Fig. 4A, increasing the MXene concentration from 0.25 to 2.0 mg·mL⁻¹ progressively enhanced the photocurrent response (444.48 nA cm^− 2^) until 0.5 mg mL^− 1^. This improvement is attributed to the conductive properties and high surface area of this nanomaterial. However, concentrations exceeding 0.5 mg·mL⁻¹ resulted in a decrease in signal due to the MXene aggregation, which formed a compact multilayer that limited electron transfer at the electrode interface [40]. The second parameter optimized was the number of voltammetric cycles used for PDA electropolymerization (5.0 mM dopamine in 100 mM PBS, pH 7.0) onto the MXene/GCE. As shown in Fig. 4B, PDA thickness was controlled by varying the number of electropolymerization cycles (5, 10, and 20 cycles), with 10 cycles yielding the optimal photocurrent response. This suggests that an adequate PDA thickness layer facilitates efficient immobilization of the cyanobacteria. Furthermore, this polymer is well known for quinone/semiquinone functional groups that act as redox mediators between the cell and the electrode surface [41]. However, 5 cycles resulted in a too-thin layer, whereas 20 cycles formed a denser film that likely restricted electron transfer and photon diffusion, thereby compromising the photocurrent.

The third parameter evaluated was the quantity of cyanobacteria immobilized on the PDA/MXene/GCE surface, assessed by adjusting the OD_680nm_ of the *S. elongatus* suspension used for photobioelectrode preparation, in Fig. 4C. The photocurrent increased with cell density up to OD_680nm_= 5.0, indicating enhanced photobiocatalytic activity and electron transfer. However, for values of OD greater than 5.0, the photocurrent response decreased, which can be attributed to excessive cell blocking of the electrode surface. This limitation may have hindered the photoactivation of PDA, thereby limiting electron transfer efficiency.

The fourth parameter optimized was the applied potential in the photobiohybrid system, using 100 mM PBS (pH 7.0). The potential range studied from 0.0 to 1.0 V is presented in Fig. 4D. The results show a dependence of current density on applied potential, with a progressive increase observed up to 0.6 V, which can be attributed to efficient charge separation induced by photosynthesis under illumination. In contrast, at potentials higher than 0.6 V, current density stabilization was observed. This behavior can be associated with saturation of the photosynthetic electron transport chain, as well as depletion of redox-active sites at the biotic–abiotic interface. Under these conditions, a potential of 0.6 V was selected for subsequent measurements. Altogether, the highest and most stable photocurrent response was achieved using 0.5 mg·mL⁻¹ of MXene, 10 electropolymerization cycles of PDA, OD_680 nm_ of 5.0, and a potential applied of 0.6 V, which was used for the next experiments.

The performance of the cyanobacteria-based photobiohybrid sensor was examined as the final parameter under different light power densities, ranging from 2.79 to 9.36 mW cm⁻² (Fig. S6). A clear correlation was observed between light intensity and current generation, with current output increasing proportionally to the rise in white-LED power density. Notably, the ability to maintain high performance even at lower light intensities highlights the photobiohybrid device’s sensitivity and efficiency, which is particularly relevant for practical applications operating under limited illumination. The results show that the photocurrent increased with power density, reaching a maximum at 9.36 mW cm⁻². Higher light intensities were not investigated due to intrinsic limitations of cyanobacterial photosynthetic machinery, as an excessive photon flux can lead to saturation and photoinhibition. These processes are often associated with increased formation of reactive oxygen species (ROS), which can ultimately restrict electron transfer efficiency [42]. Therefore, a power density of 9.36 mW cm⁻² was selected for the following experiment.

### ATZ effect on cyanobacteria-photobiohybrid sensor

**Fig. 5:**
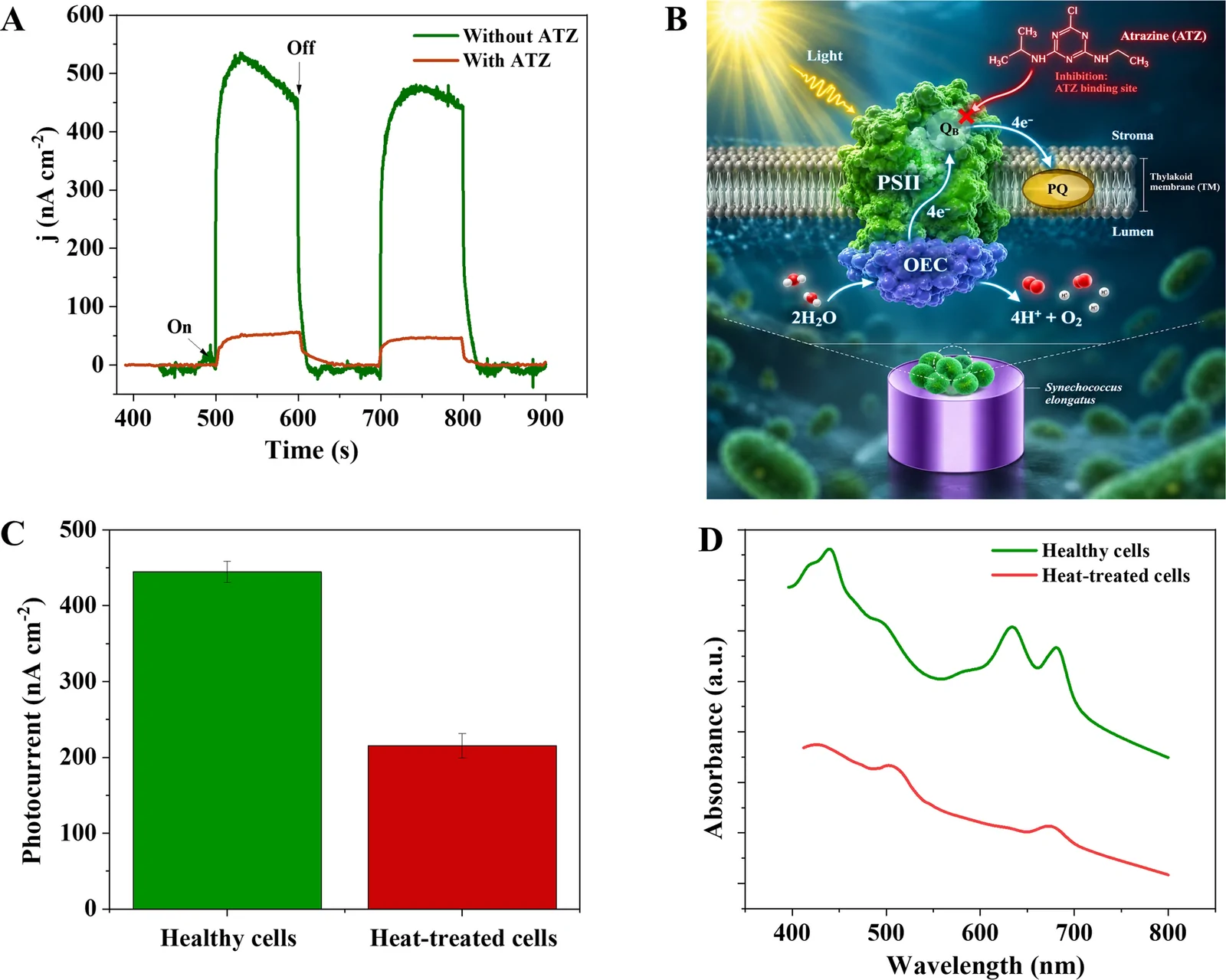
(**A**) Effect of absence (green curve) and presence of 200 nM ATZ (red curve) on the current response of a cyanobacteria-based biophotoanode. Responses at Eapp: 0.6 V in 100 mM PBS, pH 7.0. white-LED, 100 s on/off cycles. (**B**) ATZ inhibition mechanism at PSII *Q*b site, blocking electron flow from water oxidation (OEC) to PQ. The image was created using AI-generated elements from ChatGPT (https://chat.openai.com). (**C**) Comparison of the heat-treatment effect on biosensor photocurrent. Error bars represent the standard deviation (*n* = 3 different electrodes). (**D**) UV–Vis absorption spectra of healthy and thermally treated cells

The impact of the absence and presence of 200 nM ATZ on the current density of the cyanobacteria-based photobiohybrid system is shown in Fig. 5A. In the absence of herbicide, the system exhibited a stable current density. In contrast, the addition of ATZ led to a pronounced decrease in the current, reaching approximately 87%. This behavior is consistent with the well-known mode of action of triazine herbicides, which specifically target photosystem II (PSII) by binding to the 𝑄b site (the secondary plastoquinone binding pocket on the D1 protein of PSII), thereby inhibiting photosynthetic electron transport [43]. The significant suppression of the current in the presence of ATZ confirms that the electrons transferred from the cyanobacteria to the electrode surface originate predominantly from water oxidation catalyzed by PSII.

This phenomenon is explained by the known interaction of ATZ with the D1 protein (the core reaction center subunit of PSII), where it competitively binds to the *Q*b site, preventing the transfer of electrons from *Q*a (the primary plastoquinone acceptor) to plastoquinone (PQ, the mobile electron carrier in the photosynthetic electron transport chain across the thylakoid membrane, TM). As illustrated in Fig. 5B, ATZ binding at the *Q*b site (shown in red) blocks this critical step, halting the linear electron flow that normally proceeds from water oxidation at the oxygen-evolving complex (OEC), which catalyzes the reaction 2 H₂O → O₂ + 4 H⁺ + 4e⁻, to the electrode surface (arrows) [44, 45]. Consequently, the ~ 87% current suppression observed in Fig. 5A directly reflects this herbicide-induced inhibition of photogenerated electron flow, validating the photobiohybrid system’s sensitivity to PSII-targeting pollutants.

Furthermore, to avoid any potential contributions from the non-biological components of the biosensor (PDA and MXene/PDA) to the analytical response toward ATZ, control experiments were performed using electrodes prepared without cyanobacteria. As shown in Fig. S7, exposure of the control electrode to 200 nM ATZ produced no significant change in the photocurrent response, indicating that the herbicide does not appreciably affect the photoelectrochemical response of the MXene/PDA interface under the experimental conditions. These results further support that the photocurrent inhibition observed in the complete biosensor was predominantly from the interaction of ATZ with the photosynthetic machinery of *S. elongatus.*

A cell viability assay was not performed. However, to investigate the relationship between the functional state of immobilized cyanobacteria and the photoelectrochemical response, *S. elongatus* cells were subjected to heat treatment (120 °C for 2 h). As shown in Fig. 5C, heat-treated cells exhibited a decrease in photocurrent compared with untreated cells, while retaining approximately 50% of the response. This reduction supports the association between photocurrent production and the functional integrity of the photosynthetic apparatus. The residual response may arise from remaining photochemical activity in cellular components preserved after thermal treatment.

To support this interpretation, the optical absorption profiles of healthy and heat-treated cells were compared (Fig. 5D). Healthy *S. elongatus* cells exhibited the characteristic absorption features of the photosynthetic apparatus, with bands at approximately 440 and 680 nm associated with chlorophyll *a* and a band at approximately 630 nm attributed to phycocyanin [46, 47]. In contrast, thermal treatment resulted in an attenuation and loss of definition of these characteristic absorption features, consistent with disruption of the photosynthetic pigment–protein organization. These spectroscopic changes are consistent with the decreased photocurrent after thermal treatment. Phycocyanin is a major component of the phycobilisome light-harvesting antenna and contributes to excitation-energy transfer toward the chlorophyll-containing photosystems [19, 48], whereas chlorophyll *a* plays a central role in light harvesting and photochemical charge separation at the reaction centers. Disruption of these photosynthetic components is therefore expected to impair excitation-energy transfer and photosynthetic electron transport, decreasing the photoinduced electron flux available for collection at the electrode interface. The photoelectrochemical and spectroscopic results support a relationship between the functional integrity of the photosynthetic machinery and photocurrent generation.

### Analytical performance of the cyanobacteria-based biosensor for ATZ detection

**Fig. 6:**
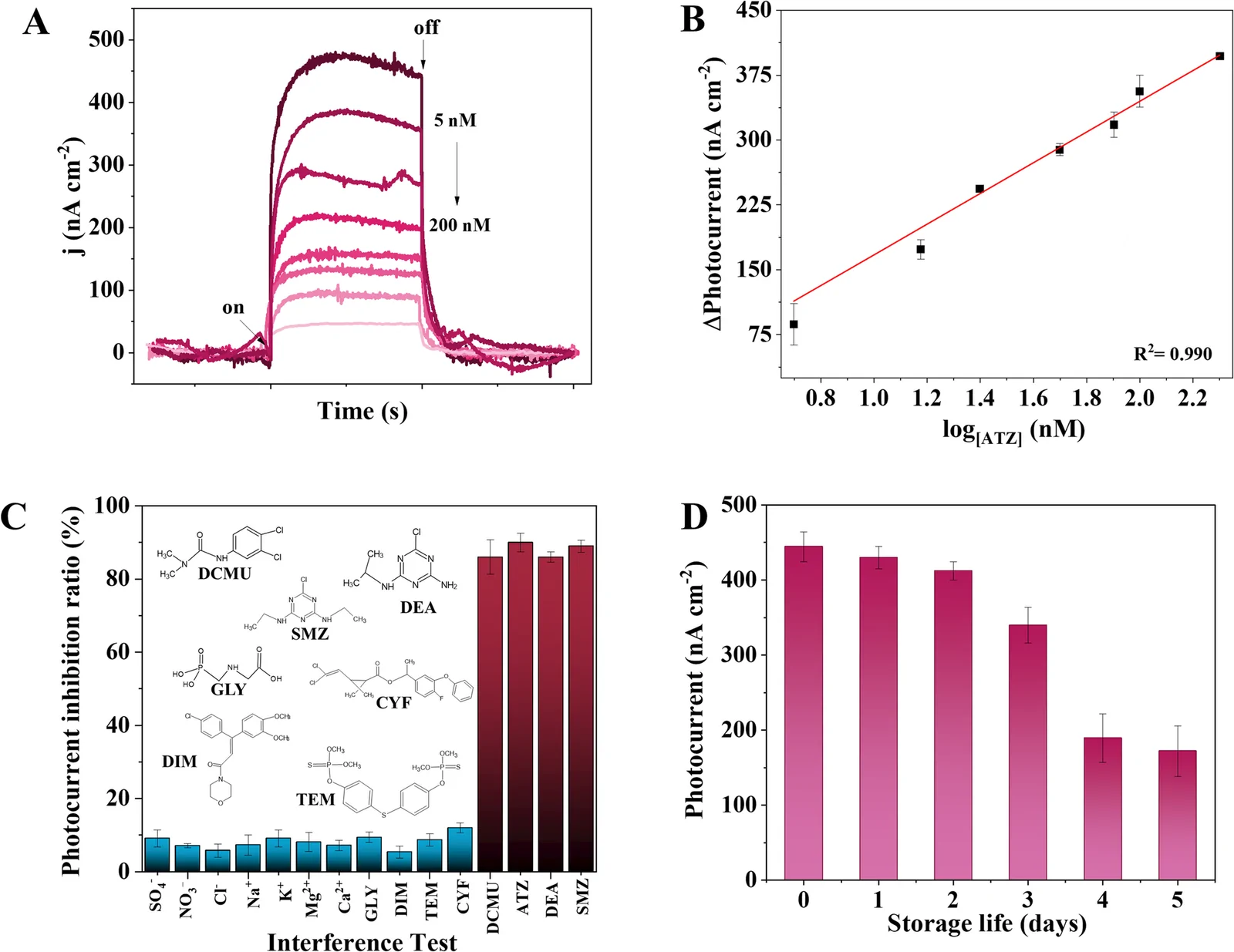
(**A**) Chronoamperometric responses in accordance with ATZ concentrations in the range of 0, 5.0, 15.0, 25.0, 50.0, 80.0, 100.0, and 200.0 nM (E_app_ =0.6 V under white LED illumination, using 100 s on/off cycles). (**B**) Analytical curve for ATZ detection using a cyanobacteria-based photobiohybrid electrode sensor. (**C**) Selectivity test of the cyanobacteria-based biosensor toward interfering ions and pesticides. (**D**) Storage life (5 days) measurements with distinct electrodes. Error bars represent standard deviation (*n* = 3 different electrodes)

Under optimal conditions, Fig. 6A shows the chronoamperometric responses of the cyanobacteria-based photobiohybrid electrode sensor to different ATZ concentrations (5.0–200 nM) in 100 mM PBS pH 7. The applied potential was 0.6 V under white LED illumination, using 100 s on/off cycles. As expected, the photocurrents decreased with increasing ATZ concentration due to inhibition of the photosynthetic machinery. Figure 6B shows the linear relationship between photocurrent and the logarithm of ATZ concentration over the studied range, with the regression equation: Photocurrent (nA cm^− 2^) = -10.82 (nA cm^− 2^) + 177.71 log_[ATZ]_ (nA cm⁻² decade⁻¹) with a coefficient of determination of 0.990. The LOD was found to be 1.92 nM (0.414 ppb), the LOQ was 5.45 nM (1.18 ppb), and the amperometric sensitivity (As) was 177.71 nA cm⁻² decade⁻¹.

To evaluate the selectivity of the developed biosensor, its response was tested in the presence of inorganic ions and different pesticide classes commonly used in agricultural and domestic environments, as shown in Fig. 6C. The studied ions included sodium (Na⁺), potassium (K⁺), magnesium (Mg²⁺), calcium (Ca²⁺), chloride (Cl⁻), sulfate (SO₄^2−^), and nitrate (NO₃⁻). In addition, the pesticides glyphosate (GLY), dimethomorph (DIM), temephos (TEM), and cyfluthrin (CYF) were analyzed. Herbicides such as 3-(3,4-dichlorophenyl)-1,1-dimethylurea (DCMU), simazine (SMZ), and desethylatrazine (DEA) were also evaluated.

The cyanobacteria-based biosensor exhibited inhibition of photocurrent after exposure to herbicides (DCMU, SMZ, and DEA), with inhibition levels approaching 100%. This behavior was expected, since these herbicides directly interfere with the photosynthetic electron transport chain at PSII, leading to a decrease in the generated photocurrent [44]. In contrast, the other pesticides investigated (DIM, CYF, TEM, and GLY) and ions, despite their widespread use in agricultural and domestic pest control, showed no significant interference (< 10% of the analyte signal) [49], possibly due to their distinct modes of action, which do not directly impact the photosynthetic processes responsible for photocurrent generation in the cyanobacteria-based system. These findings demonstrate that the biosensor is selective toward PSII-targeting herbicides rather than being specific to atrazine alone.

From an environmental monitoring perspective, the biosensor is particularly suitable as a rapid screening tool for the presence of PSII-inhibiting herbicides in environmental waters.

To evaluate storage stability, biosensors were stored at 4 °C and tested over 5 days in the absence of ATZ, as illustrated in Fig. 6D. The photocurrent responses remained > 95% for 1 day and approximately 93% at day 2 (RSD < 3.8%). After the third day, the response began to decline, reaching approximately a 61% decrease by the fifth day. This behavior can be attributed to the inherent biological limitations of cyanobacteria-based systems, including metabolic stress and partial loss of photosynthetic activity.

The reproducibility of the fabrication procedure was evaluated using five independently prepared cyanobacteria-based biosensors. Each electrode was fabricated following the complete modification and cell-immobilization protocol described in the experimental section and subsequently evaluated under identical photoelectrochemical conditions. An RSD of 4.60% was obtained for the photocurrent response (*n* = 5), indicating good reproducibility.

 In Table S2, the analytical performance of biosensors reported in the literature for ATZ detection is compared to that of the biosensor proposed in this work. The analytical performance of electrochemical biosensors reported in the literature for ATZ detection covers different biological recognition elements as well as different transduction techniques. It can be observed that in enzymatic systems, the use of tyrosinase with electrochemiluminescence (ECL) detection stands out, achieving an LOD of 3.71 × 10⁻¹³ mol L⁻¹ [50], representing one of the lowest LODs in this category. However, the fabrication of these devices generally requires additional enzyme immobilization steps and exhibits limited operational stability. The LODs obtained for antibody-based immunosensors ranged from 10⁻¹⁵ to 10⁻²¹ mol L⁻¹ [51, 52], with impedimetric configurations standing out for reaching ultrasensitive detection levels, but they often demand laborious labeling and purification procedures, in addition to presenting high costs. Biosensors based on aptamers and DNA have high affinity and specificity and excellent sensitivity, presenting LODs in the range of 10⁻¹⁴ to 10⁻¹⁹ mol L⁻¹ [53–56], especially when featuring impedimetric detection, demonstrating excellent performance in complex matrices. Additionally, Table S2 reveals a wide diversity of photosynthetic units and electrode architectures, with LODs typically ranging from 10⁻^6^ to 10⁻^9^ mol L⁻¹ [57–60]. Despite these advances, many of these sensors present limitations regarding bioelement stability, response time, and reproducibility. In comparison, the biosensor proposed in this work, based on *S. elongatus* cyanobacteria immobilized on an electrode modified with MXene and PDA, achieved an LOD of 1.92 × 10⁻^9^ mol L⁻¹ (0.414 ppb), with a linear range from 5.00 × 10⁻⁹ to 2.00 × 10⁻⁷ mol L⁻¹ and successful application in environmental water samples. Although this LOD value is higher than that of some more sensitive aptamers and immunosensors, it proves to be comparable to or better than most photosynthetic biosensors reported with intact cells, especially considering operational simplicity, low cost, biocompatibility, and rapid response capability. Furthermore, the obtained LOD is below the maximum allowable limit established by EPA legislation (3.0 ppb) for drinking water, demonstrating its suitability for practical environmental monitoring applications. Overall, the proposed configuration establishes a new MXene-based photobiohybrid sensing strategy and represents a promising alternative for environmental monitoring of ATZ at low concentrations.

### Detection of ATZ in water samples

To validate the practical applicability of the proposed biosensor, recovery experiments were conducted using river, groundwater, and seawater samples. Known amounts of ATZ were spiked into the samples at a concentration of 80 nM, and the recovered concentrations were determined by interpolation from the analytical curve. As summarized in Table 1, the recovery values ranged from 99.75% to 103.9%, with relative standard deviations (RSD) between 2.8% and 4.80%. These results demonstrate that the developed biosensor enables accurate and reliable determination of ATZ in complex environmental water matrices. In addition, to further validate the performance of the proposed biosensor, the real samples were also analyzed by LC–MS/MS, and these comparative results have been incorporated into the Table.

**Table 1:** ATZ concentration recovered from river, groundwater, and seawater samples using the cyanobacteria-based biosensor and LC-MS/MS

Sample	Cyanobacteria-based Biosensor			LC-MS/MS		
	[ATZ]_found (nM)_	Rec. (%)	RSD (%)	[ATZ]_found (nM)_	Rec. (%)	RSD (%)
River	82.0	102.5	2.8	78.8	98.5	1.25
Groundwater	79.8	99.75	4.5	80.2	100.2	1.86
Seawater	83.1	103.9	4.8	78.5	98.1	2.17

### Greenness and practical applicability assessment of the cyanobacteria-based biosensor and LC-MS/MS methods

**Fig. 7:**
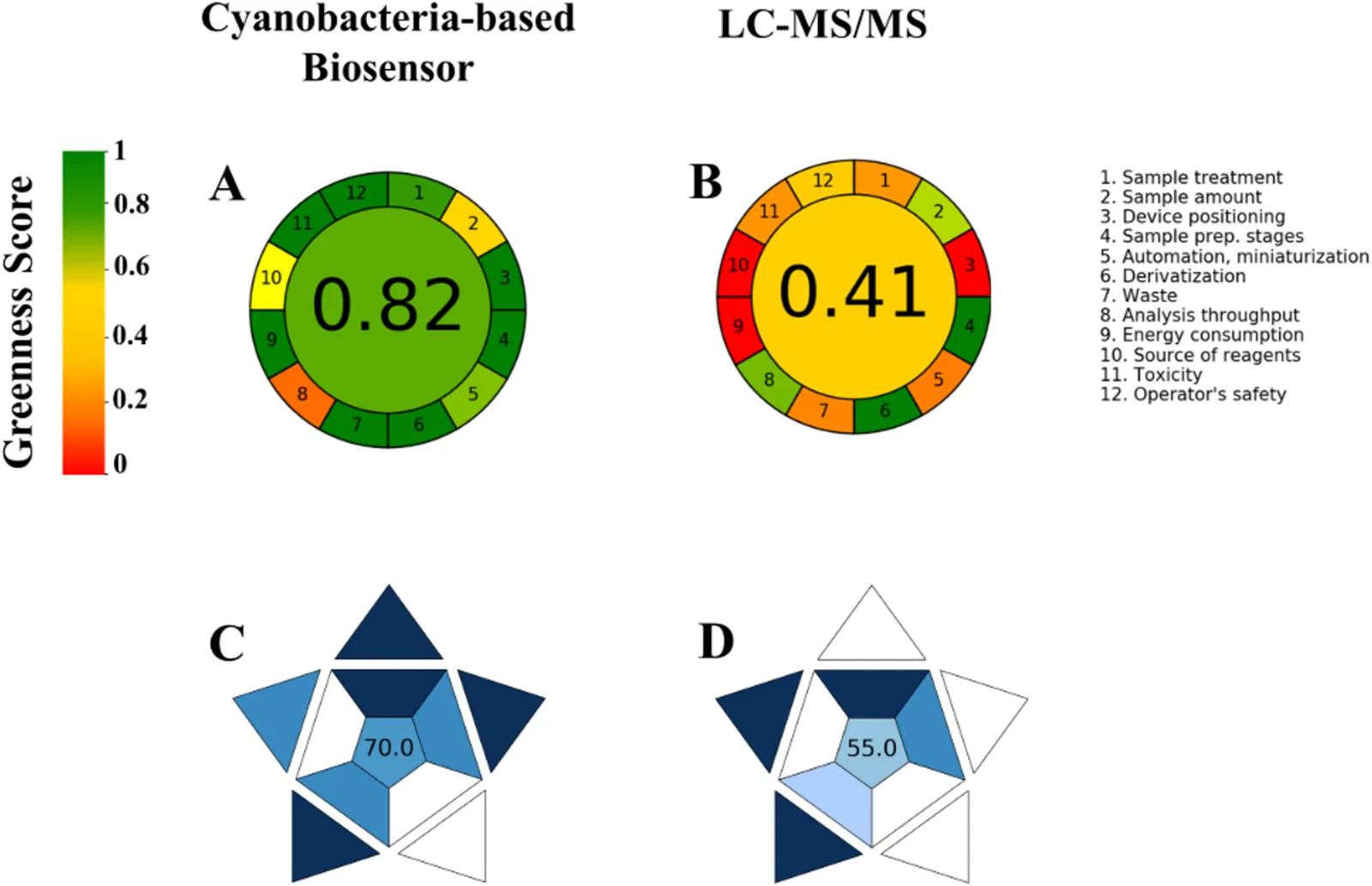
AGREE and BAGI scores obtained for the cyanobacteria-based biosensor (**A**-**C**) and LC-MS/MS (**B**-**D**), confirming the eco-friendly, sustainability, practicality, and efficiency characteristics of the device

The greenness and practical applicability of the proposed cyanobacteria-based biosensor were evaluated using the complementary AGREE [61] and BAGI [62] metrics and compared with LC-MS/MS, used as the reference method for ATZ determination, as shown in Fig. 7. AGREE evaluates compliance with the principles of Green Analytical Chemistry on a scale from 0 to 1, whereas BAGI assesses practical aspects of the analytical procedure on a scale from 0 to 100, with higher scores representing more favorable performance in both metrics.

The cyanobacteria-based biosensor achieved an AGREE score of 0.82, substantially higher than the 0.41 obtained for LC-MS/MS. This difference reflects favorable characteristics of the proposed method, particularly reduced reagent and energy consumption, limited waste generation, and simplified sample preparation. The BAGI assessment showed a similar behaviour, with scores of 70 for the biosensor and 55 for LC-MS/MS, indicating greater practical applicability of the proposed approach under the evaluated criteria. Nevertheless, the combined AGREE and BAGI results demonstrate that the biosensor provides a more favorable greenness and applicability profile than chromatographic analysis under the evaluated conditions. Importantly, this advantage was achieved while maintaining satisfactory analytical performance in environmental water samples, as demonstrated by the recovery results obtained in comparison with LC-MS/MS.

## Conclusions

In this work, a photobioelectrochemical biosensor was developed by immobilizing whole *S. elongatus* cells onto a Ti₃C₂Tₓ MXene electrode modified with electropolymerized PDA. The conductive MXene/PDA interface established an efficient biotic–abiotic pathway, enhancing light-driven electron extraction from cyanobacteria under visible light.

The sensing mechanism relies on the inhibition of PSII activity. Rather than being specific to ATZ, the biosensor functions as a class-selective screening device for PSII-inhibiting herbicides (e.g., ATZ, SMZ, DCMU, desethylatrazine), with minimal interference (≤ 12%) from non-PSII pesticides and inorganic ions. Using ATZ as an analyte, the platform achieved a LOD of 1.92 nM (0.414 ppb), a linear range of 5–200 nM, and accurate recoveries (99.75–103.9%) in environmental water samples, validated by LC-MS/MS.

Regarding the stability, the biosensor retained > 95% for 1 day and approximately 93% at day 2 when stored at 4 °C. However, a 61% decrease in photocurrent occurred by day 5, representing a primary limitation of the platform due to the loss of cellular viability and photosynthetic integrity over time. Despite this stability limitation, high AGREE (0.82) and BAGI (70.0) scores confirm that the proposed device provides an eco-friendly, rapid, and low-cost alternative for screening PSII-inhibiting herbicides.

## Supplementary Information

Below is the link to the electronic supplementary material.

Supplementary Material 1 (DOCX 1.22 MB)

## Data Availability

No datasets were generated or analysed during the current study.

## Declarations

### AI usage statement

Artificial intelligence (AI) tools were used exclusively for language refinement, including grammar correction, improvement of language clarity, and enhancement of overall readability, as well as for the creation of graphic elements presented in Fig. 5B and the Graphical Abstract. No AI tools were used for the generation of scientific content, data analysis, interpretation of results, formulation of conclusions, or critical revision of the manuscript. All scientific aspects of the work were conducted solely by the authors, who assume full responsibility for the accuracy, originality, integrity, and validity of the research and its findings.
