# Supplementary material for: MXene-polydopamine-cyanobacteria photobioelectrochemical biosensor for atrazine detection in environmental waters
Source: Mikrochim Acta. 2026 Oct 23;193(11):752. doi: 10.1007/s00604-026-08471-z (PMC13694299; doi:10.1007/s00604-026-08471-z)
Supplement: Supplementary file 1 [file 604_2026_8471_MOESM1_ESM.docx]

**Supporting Information**

**MXene-Polydopamine-Cyanobacteria Photobioelectrochemical Biosensor for Atrazine Detection in Environmental Waters**

**Caio César Gonçalves Silva*^b,1^, Maiza Lima Bueno^a1^, Max Fabricio Falone^b^, Lucas Felipe de Lima^c^, William Reis de Araujo^c^, Maria Valnice Boldrin Zanoni^b^, Maisa Azevedo Beluomini*^a,b^**

^a^ Universidade Estadual Paulista (UNESP), Faculdade de Ciências Agrárias e Veterinárias, Jaboticabal, São Paulo, Brasil.

^b^ Universidade Estadual Paulista (UNESP), Instituto de Química, Araraquara, São Paulo, Brasil.

^c^ Departamento de Química Analítica, Instituto de Química, Universidade Estadual de Campinas (UNICAMP), Campinas, São Paulo, Brasil.

**^1^** These authors contributed equally to this work.

***Corresponding author:** ccg.silva@unesp.br; maisa.beluomini@unesp.br

**Table of Content**

Synthesis and characterization of MXene	Page S3

Figure S1	Page S4

Ti₃AlC₂ and Ti₃C₂Tₓ Characterization	Page S5

Figure S2	Page S7

Figure S3	Page S8

Figure S4	Page S9

Figure S5	Page S10

Table S1	Page S11

Figure S6	Page S12

Figure S7	Page S13

Table S2	Page S14

References to the Supporting Information	Page S18

**Synthesis of MXene**

The synthesis of the MXene nanomaterial was based on a previously established protocol described in the literature [1, 2]. Initially, 1.0 g of lithium fluoride (LiF) was carefully dissolved in 20 mL of 6.0 M hydrochloric acid (HCl). Subsequently, 1.0 g of Ti₃AlC₂ (MAX phase) was gradually added to the LiF/HCl mixture under vigorous stirring in a Teflon vessel maintained in a 35 °C water bath. The reaction mixture was continuously stirred at this temperature for 24 hours. The resulting suspension was centrifuged at 3500 rpm for 15 min, washed multiple times with ultrapure water, and the pH of the supernatant was adjusted to 7.0. The material was dried at 60 °C and stored in plastic containers until further use.

**Characterization of MXene**

Fourier Transform Infrared (FTIR) and X-ray Photoelectron Spectroscopy (XPS) characterization of the material is shown in Fig. S2-3. NAP-XPS measurements were performed on a commercial spectrometer (EnviroESCA, SPECS, Germany). equipped with a PHOIBOS 150 NAP analyzer and a 1D-DLD detector. Monochromatic Al Kα radiation (1486.71 eV) was used as the X-ray source. with a nominal beam size of 300 µm. The analyzer was operated with an entrance slit of 7 × 20 mm. an exit slit configured with a mesh. and an iris/aperture of 60 mm. The inelastic background of the high-resolution spectra (C 1s. O 1s. F 1s. and Ti 2p) was subtracted based on the Shirley method.

**Fig. S1.** Growth curve of the cyanobacteria S. elongatus monitored over time under controlled culture conditions. The inset shows an image of the cyanobacterial culture after 48 h of growth period. Data represent mean ± standard deviation (n = 3).

**Ti₃AlC₂ and Ti₃C₂Tₓ Characterization**

Fig. S2 A, acquired at higher magnification, exhibits the characteristic 3D wrinkled flake architecture of Ti₃C₂Tₓ, consistent with literature reports [3, 4]**.** FTIR spectroscopy was employed to characterize the MXene material (Fig. S2 B). In its fingerprint region (400–1400 cm⁻¹), characteristic absorption bands were observed, including a Ti–O bending vibration around 590 cm⁻¹ and a Ti–C stretching vibration near 600 cm⁻¹ [5]. The material also exhibits a C–OH bending band near 1380 cm⁻¹, indicative of tertiary alcohol groups, as well as a band around 1630 cm⁻¹ corresponding to aromatic C=C stretching and O–H bending [6]. These spectral features support the formation of MXene with the chemical formula Ti₃C₂Tₓ, where T represents surface terminations, primarily oxygen. Additionally, a broad O–H stretching band in the 3200–3600 cm⁻¹ range was detected, which is attributed to the presence of confined water in the KBr pellet resulting from the sample preparation process [7]. For comparison, the FTIR of the precursor Ti₃AlC₂ is shown in the red line.

To confirm the synthesis, high-resolution XPS spectra of the C 1s, Ti 2p, O 1s, and F 1s regions were acquired to examine the surface composition and chemical environments of the synthesized Ti₃C₂Tₓ MXene. The C 1s spectrum (Fig. S3 A) contains a low-binding-energy component at approximately 281.9 eV, characteristic of the carbide C-Ti bonds forming the MXene backbone, together with contributions associated with surface-modified C-Ti-Tₓ environments, adventitious CC/C-H, and oxygenated carbon species. The Ti 2p envelope was fitted using physically constrained Ti 2p₃/₂-Ti 2p₁/₂ doublets, with spin-orbit separations of approximately 5.7-6.1 eV and area ratios close to 2:1 (Fig. S3 B). The component near 455.6 eV is assigned to carbide Ti-C. In contrast, the contributions between approximately 457 and 458 eV are associated with different Ti-C-Tₓ environments involving O- and OH-containing surface terminations [8]. A higher-binding-energy contribution near 459.5 eV indicates partial formation of TiOₓ/TiO₂like species at the MXene surface [9, 10]. In the O 1s region (Fig. S3 C), the component near 530.7 eV is attributed primarily to Ti-O environments, while the higher-binding-energy contributions arise from surface O/OH terminations and adsorbed oxygenated species [11]. In contrast, the F 1s components observed at approximately 687.3 and 689.3 eV occur above the characteristic range reported for MXene-bound Ti-F species, typically located near 684.5–685.5 eV, and are therefore assigned predominantly to fluorocarbon surface contamination [9, 12–14] (Fig. S3 D). Overall, the XPS results support the formation of the Ti₃C₂Tₓ carbide framework and its oxygen-containing surface chemistry, while also indicating partial surface oxidation.

The electrochemical effect of MXene modification, under optimal conditions, was further investigated by CV using the [Fe(CN)₆]³⁻/⁴⁻ redox probe. As shown in Fig. S4, the MXene/GCE exhibited higher anodic and cathodic peak currents compared with the bare GCE, demonstrating an improvement in the electrochemical response after surface modification. This behavior can be attributed to the high electrical conductivity and favorable interfacial properties of Ti₃C₂Tₓ MXene, which facilitate charge transport at the electrode/electrolyte interface. These results are consistent with the EIS measurements, Fig. S5, for which the charge-transfer resistance decreased from 0.34 kΩ for the bare GCE to 0.21 kΩ after MXene modification. Accordingly, the heterogeneous electron-transfer rate constant (k⁰) increased from 2.21 × 10⁻⁶ to 3.59 × 10⁻⁶ cm s⁻¹. Taken together, these results provide complementary evidence that incorporation of MXene improves the interfacial electron-transfer properties of the GCE, supporting its role as a conductive component in the proposed photobioelectrochemical architecture.

**Fig. S2.** (A) FEG-SEM of MXene with 30,000x magnification, and (B) FTIR spectra of MXene (black line) and the precursor Ti₃AlC₂ (red line).

**Fig. S3:** High-resolution XPS spectra deconvoluted for: (A) C1s, (B) Ti 2p, (C) O 1s, (D) F 1s.

**Fig. S4.** Cyclic voltammograms of bare GCE (blue curve) and MXene-modified GCE (pink curve). Experimental conditions: 5.0 mM of [Fe(CN)₆]³⁻/⁴⁻ as a redox probe in 0.1 M KCl at 50 mV s^-1^.

**Fig. S5.** Nyquist diagrams of EIS of different electrodes (a) bare GCE (b) MXene/GCE (c) PDA/MXene/GCE, (d) *S.elongatus*/PDA/Mxene/GCE. The supporting solution contained 5.0×10^−3^ mol L^−1^ [Fe(CN)_6_]^3-/4-^ and 0.1 mol L^−1^ KCl and the frequency range was of 0.01 Hz to 100 kHz. Inset: equivalent circuit.

**Table S1.** *R_ct_* and *k*^0^ values calculated from Nyquist plots (Fig. S5) under light conditions.

| **Electrode** | ***R_ct_* (kΩ)** | ***k*^0^ (cm s^-1^)** |
| --- | --- | --- |
| GCE | 0.34 | 2.21× 10⁻⁶ |
| MXene | 0.21 | 3.59 × 10⁻⁶ |
| PDA-MXene | 0.78 | 9.66 × 10⁻⁷ |
| *S.elongatus*/PDA/Mxene/GCE | 4.97 | 1.51 × 10⁻⁷ |

**Fig. S6.** Impact of white-LED illumination intensity on the photocurrent response for the cyanobacteria photobioelectrode. Electrolyte: 100 mM PBS, pH 7.0; Eapp: +0.6 V vs. Ag/AgCl/3.0 M KCl. S. elongatus (OD₆₈₀ = 5.0). Error bars represent standard deviation (n = 3).

**Fig. S7.** Control experiments PDA and MXene-PDA evaluating the contribution of the biological component to the absence and presence of 200 nM of ATZ sensing response. Error bars represent the standard deviation (n = 3 different electrodes). Experimental conditions: 100 mM PBS (pH 7.0); Eapp: 0.6 V vs. Ag/AgCl (3.0 M KCl).

**Table S2.** Comparison of the analytical performance of biosensors for ATZ detection.

| **Biological Receptor** | | **Electrochemical Detection** | **Concentration range (mol L^-1^)** | **LOD (mol L^-1^)** | **Samples** | **Ref** |
| --- | --- | --- | --- | --- | --- | --- |
| **Enzymes** | Tyrosinase | Amperometric | 6.6 × 10⁻⁶ – 4.50 × 10⁻⁵ | 1.39 × 10⁻⁶ | Lake water | [15] |
|  | Carboxylesterase | DPV | 1.00 × 10^−6^ –4.00 × 10^−4^ | 5.4 × 10⁻⁶ | Wastewater, Tap water, and milk | [16] |
|  | Tyrosinase | Amperometric | 2.32 × 10⁻⁷ – 1.39 × 10⁻⁴ | 4.64 × 10⁻⁸ | River water | [17] |
|  | GOx | SWV | 5.80 × 10^−7^– 4.22 × 10^−5^ | 3.90 × 10⁻⁸ | Wastewater | [18] |
|  | HRP | Amperometric | 1.00 × 10⁻⁵ – 4.00 × 10⁻⁵ | 1.60 × 10⁻^6^ | n.d. | [19] |
|  | Tyrosinase | ECL | 4.64 × 10⁻¹³ – 9.27 × 10⁻⁸ | 3.71 × 10⁻¹³ | River, Tap, and Lake water | [20] |
| **Antibody** | Antibody | Conductometric | 1.48 × 10⁻^9^ – 9.27 × 10⁻⁶ | **1.58 × 10⁻^10^** | Red wine | [21] |
|  | Antibody | CV | 4.64 × 10⁻¹¹ – 4.64 × 10⁻⁶ | 5.56 × 10⁻⁹ | Territorial water, soil, corn flakes, poultry, and bovine feeds | [22] |
|  | Antibody | DPV | 1.85 × 10⁻¹⁰ – 2.78 × 10⁻⁹ | 8.35 × 10⁻¹¹ | n.d. | [23] |
|  | Antibody | DPV | 2.32 × 10⁻¹¹ – 2.32 × 10⁻⁶ | 7.00 × 10⁻¹¹ | Maize | [24] |
|  | Antibody | DPV | 2.32 × 10⁻¹⁰ – 6.95 × 10⁻⁹ | 5.10 × 10⁻¹¹ | Drinking water | [25] |
|  | Antibody | SWV | 9.27 × 10⁻¹² – 9.27 × 10⁻⁸ | 4.30 × 10⁻¹¹ | n.d. | [26] |
|  | Antibody | Impedimetric | 4.64 × 10⁻¹¹ – 2.32 × 10⁻⁷ | 4.64 × 10⁻¹¹ | n.d. | [27] |
|  | Antibody | FFT-SWV | 5.00 × 10⁻¹⁰ – 1.00 × 10⁻⁷ | 2.00 × 10⁻¹¹ | n.d. | [28] |
|  | Antibody | Conductimetric | 1.00 × 10⁻¹³ – 1.00 × 10⁻⁵ | 9.97 × 10⁻¹² | Purified water | [29] |
|  | Antibody | SWV | 1.00 × 10⁻¹² – 1.00 × 10⁻⁵ | 9.27 × 10⁻¹³ | n.d. | [30] |
|  | Antibody | Amperometric | 9.27 × 10⁻¹³ – 9.27 × 10⁻¹¹ | 9.27 × 10⁻¹³ | River water | [31] |
|  | Antibody | DPV | 2.32×10^−10^ – 1.39×10^−7^ | 3.71 × 10⁻¹³  2.78 × 10⁻¹³ | Groundwater, plum juice, and rice extract | [32] |
|  | Antibody | Impedimetric | **2.32 × 10⁻⁹ – 4.64 × 10⁻⁵** | 4.64 × 10⁻¹⁵ | n.d. | [33] |
|  | Antibody | Impedimetric | 1.00 × 10⁻²⁰ – 1.00 × 10⁻⁶ | 5.37 × 10⁻²¹ | n.d. | [34] |
| **Nucleotide (Aptamer and DNA)** | Aptamer | Amperometric | 1.00 × 10⁻⁷ – 2.00 × 10⁻⁷ | 7.50 × 10⁻⁹ | Domestic sewage | [35] |
|  | [Aptamer](https://www-sciencedirect-com.ez87.periodicos.capes.gov.br/topics/biochemistry-genetics-and-molecular-biology/aptamer) | Impedimetric | 1.00 × 10^−10^ – 1.00 × 10^−6^ | 1.00 × 10⁻¹¹ | Tap and mineral water | [36] |
|  | Aptamer | Amperometric | 1.00 × 10⁻¹¹ – 5.00 × 10⁻⁸ | **5.40 × 10⁻¹²** | Lake water | [37] |
|  | Aptamer | Impedimetric | 4.64 × 10⁻¹² – 2.32 × 10⁻⁷ | 3.11 × 10⁻¹² | River and Tap water | [38] |
|  | Aptamer | CA | 5.00 × 10⁻¹² – 1.00 × 10⁻⁸ | 1.20 × 10⁻¹² | Lake water | [39] |
|  | Aptamer | PEC | 2.50 × 10⁻¹² – 5.00 × 10⁻¹⁰ | 1.00 × 10⁻¹² | Lake, Wastewater, and sewage water | [40] |
|  | Aptamer | CA | 2.32 × 10⁻¹^2^ – 1.39 × 10⁻^8^ | 7.74 × 10⁻¹³ | River water | [41] |
|  | DNA | Amperometric | 9.27 × 10⁻⁹ – 4.64 × 10⁻⁶ | 3.47 × 10⁻¹³ | Tomato, apple, and river water | [42] |
|  | Aptamer | DPV | 2.50 × 10⁻¹³ – 2.50 × 10⁻¹⁰ | 1.00 × 10⁻¹³ | Lake and river water | [43] |
|  | Aptamer | Amperometric | 1.00 × 10⁻¹² – 1.00 × 10⁻⁷ | **1.00 × 10⁻¹³** | River and tap water | [44] |
|  | Aptamer | Impedimetric | 1.85 × 10⁻¹³ – 1.85 × 10⁻⁸ | 2.27 × 10⁻¹⁴ | Corn | [45] |
|  | Aptamer | Amperometric | 5.00 × 10⁻¹⁴ – 3.00 × 10⁻¹⁰ | 1.20 × 10⁻¹⁴ | Lake water | [46] |
|  | [Aptamer](https://www-sciencedirect-com.ez87.periodicos.capes.gov.br/topics/biochemistry-genetics-and-molecular-biology/aptamer) | Amperometric | 4.64 × 10⁻¹⁴ – 4.64 × 10⁻⁸ | 1.07 × 10⁻¹⁴ | corn | [47] |
|  | Aptamer | Amperometric | 1.00 × 10⁻¹⁶ – 1.00 × 10⁻⁹ | 3.33 × 10⁻¹⁷ | Tap, and river water | [48] |
|  | DNA | DPV | 1.0 × 10^−18^ – 1.0 × 10^−12^ | 3.40 × 10⁻¹⁹ | Groundwater, Lake, River, and Stream water | [49] |
| **Photosynthetic Units** | *Synechocystis* PCC6803 | Amperometric | n.d | n.d | n.d | [50] |
|  | *C. reinhardtii* | Amperometric | 1.00 × 10⁻⁷ – 6.60 × 10⁻⁶ | 1.99 × 10⁻⁶ | River water | [51] |
|  | *C. vulgaris* | Amperometric | 2.50 × 10⁻⁷ – 1.00 × 10⁻⁴ | 1.00 × 10⁻⁶,  5.01 × 10⁻⁸ | n.d | [52] |
|  | *C. vulgaris* | Amperometric | 5.00 × 10⁻⁸ – 1.00 × 10⁻⁴ | 9.97 × 10⁻⁷ | n.d | [53] |
|  | *C. pyrenoidosa* | Amperometric | 9.00 × 10⁻⁷ – 7.40 × 10⁻⁵ | 7.00 × 10⁻⁷ | river water and bottled mineral water | [54] |
|  | *C. vulgaris* | Amperometric | 1.00 × 10⁻⁷ – 1.00 × 10⁻⁶ | 2.32 × 10⁻⁷ | n.d | [55] |
|  | *S. acutus* / *M. contortum* | Amperometric | **1.00 × 10⁻⁶ – 3.00 × 10⁻⁵** | **1.11 × 10⁻⁷** | River water | [56] |
|  | [*Chlamydomonas reinhardtii*](https://www-sciencedirect-com.ez87.periodicos.capes.gov.br/topics/agricultural-and-biological-sciences/chlamydomonas-reinhardtii) | Amperometric | 6.00 × 10^−9^ – 9.00 × 10^−8^ | 9.00 × 10⁻⁸ | n.d. | [57] |
|  | *A. variabilis* | Amperometric | 1.80 × 10⁻⁷ – 6.18 × 10⁻⁶ | 6.95 × 10⁻⁸ | n.d | [58] |
|  | *Paulschulzia pseudovolvox* | Amperometric | 1.00× 10⁻⁷ – 1.20 × 10⁻⁶ | 2.40 × 10⁻⁸ | Tap water | [59] |
|  | *Leptolyngbia* sp. | Amperometric | 2.00 × 10⁻⁷ – 8.00× 10⁻⁷ | 2.40 × 10⁻⁸ | Tap water | [60] |
|  | PSII | Amperometric | 1.00 × 10⁻⁹ – 1.00 × 10⁻⁶ | 2.00 × 10⁻⁹  4.00 × 10⁻⁹ | n.d. | [61] |
|  | RC | Amperometric | 2.08 × 10⁻⁷ – 2.10 × 10⁻⁶ | **5.01 × 10⁻⁸**  8.81 × 10⁻⁹ | n.d | [62] |
|  | Thylakoid membrane | Amperometric | 1.00 × 10⁻⁷ – 1.20 × 10⁻⁶ | 9.50 × 10⁻⁹ | Tap water | [63] |
|  | *Paulschulzia pseudovolvox* | Amperometric | 1.00 × 10⁻⁷ – 1.20 × 10⁻⁶ | 7.50 × 10⁻⁹ | Tap water | [64] |
|  | Thylakoid membranes | Amperometric | 1.72 × 10⁻⁹ – 5.56 × 10⁻⁸ | 1.72 × 10⁻⁹ | n.d. | [65] |
|  | PSII | Amperometric | 1.00 × 10⁻¹⁰ – 1.00 × 10⁻⁴ | 1.62 × 10⁻⁹ | n.d. | [66] |
|  | PSII | Amperometric | 1.00 × 10⁻⁹ – 1.00 × 10⁻⁶ | 1.15 × 10⁻⁹. | n.d | [67] |
|  | *Chlamydomonas reinhardtii* | Amperometric | 1.00 × 10⁻⁷ – 5.00 × 10⁻⁶ | 1.10 × 10⁻^9^ | Wastewater | [68] |
|  | *Chlamydomonas reinhardtii* | Amperometric | 1.00 × 10⁻⁷ – 5.00 × 10⁻⁶ | 1.00 × 10⁻⁹ | River water | [69] |
|  | *S. elongatus* | Amperometric | **5.00 × 10⁻⁹ – 2.00 × 10⁻⁷** | 1.92 × 10⁻^9^ | River water, Groundwater and Seawater | This work |

**References**

1.	de Lima LF, Ferreira AL, dos Santos LE, et al (2025) Bacterial Cellulose-Based Laser-Scribed Graphene Electrode for Hydrogen Peroxide Detection in Cancer Cells. ACS Appl Bio Mater 8:6339–6349. https://doi.org/10.1021/acsabm.5c00825

2.	Liu F, Han L, Yang Y, et al (2023) Designable synthesis of a novel layered MXene loaded gold nanocluster composite for efficient electrochemical sensing of homocysteine in biological samples. Chemical Engineering Journal 461:141928. https://doi.org/10.1016/j.cej.2023.141928

3.	Alnoor H, Elsukova A, Palisaitis J, et al (2021) Exploring MXenes and their MAX phase precursors by electron microscopy. Materials Today Advances 9:100123. https://doi.org/10.1016/j.mtadv.2020.100123

4.	Zhao D, Clites M, Ying G, et al (2018) Alkali-induced crumpling of Ti3C2Tx (MXene) to form 3D porous networks for sodium ion storage. Chem Commun 54:4533–4536. https://doi.org/10.1039/C8CC00649K

5.	VahidMohammadi A, Moncada J, Chen H, et al (2018) Thick and freestanding MXene/PANI pseudocapacitive electrodes with ultrahigh specific capacitance. J Mater Chem A 6:22123–22133. https://doi.org/10.1039/C8TA05807E

6.	Dai F, Zhuang Q, Huang G, et al (2023) Infrared Spectrum Characteristics and Quantification of OH Groups in Coal. ACS Omega 8:17064–17076. https://doi.org/10.1021/acsomega.3c01336

7.	Parker T, Zhang D, Bugallo D, et al (2024) Fourier-Transform Infrared Spectral Library of MXenes. Chem Mater 36:8437–8446. https://doi.org/10.1021/acs.chemmater.4c01536

8.	Näslund LÅ, Persson I (2022) XPS spectra curve fittings of Ti3C2Tx based on first principles thinking. Applied Surface Science 593:153442. https://doi.org/10.1016/j.apsusc.2022.153442

9.	Halim J, Cook KM, Naguib M, et al (2016) X-ray photoelectron spectroscopy of select multi-layered transition metal carbides (MXenes). Applied Surface Science 362:406–417. https://doi.org/10.1016/j.apsusc.2015.11.089

10.	Ni M, Chen J, Wang C, et al (2022) A high-sensitive dopamine electrochemical sensor based on multilayer Ti3C2 MXene, graphitized multi-walled carbon nanotubes and ZnO nanospheres. Microchemical Journal 178:107410. https://doi.org/10.1016/J.MICROC.2022.107410

11.	Benchakar M, Loupias L, Garnero C, et al (2020) One MAX phase, different MXenes: A guideline to understand the crucial role of etching conditions on Ti3C2Tx surface chemistry. Applied Surface Science 530:147209. https://doi.org/10.1016/J.APSUSC.2020.147209

12.	Natu V, Benchakar M, Canaff C, et al (2021) A critical analysis of the X-ray photoelectron spectra of Ti3C2Tz MXenes. Matter 4:1224–1251. https://doi.org/10.1016/j.matt.2021.01.015

13.	Nguyen L, Ali A, Aissa B, et al (2025) Electrochemical investigation of Ti3C2Tx (MXene), N-Ti3C2Tx, and the Ti3C2Tx/Co3O4 hybrid composite deposited on carbon cloth for use as anode materials in flexible supercapacitors. Nanoscale Advances 7:5313–5322. https://doi.org/10.1039/d4na01024h

14.	Falone MF, Olsen BG, Buffon E, Stradiotto NR (2024) Electrochemical sensing of palmitic acid in guava oil using a surface modified with reduced graphene oxide-decorated manguanese nanoparticles and electropolymerized poly(serine) film. Journal of Food Composition and Analysis 135:106550. https://doi.org/10.1016/j.jfca.2024.106550

15.	Tortolini C, Bollella P, Antiochia R, et al (2016) Inhibition-based biosensor for atrazine detection. Sensors and Actuators B: Chemical 224:552–558. https://doi.org/10.1016/j.snb.2015.10.095

16.	Singh S, N. P, Kaur H, et al (2024) Enzyme-based sensor for the real-time detection of atrazine: Evidence from electrochemical and docking studies. Sci Rep 14:17662. https://doi.org/10.1038/s41598-024-65801-y

17.	Guan Y, Liu L, Chen C, et al (2016) Effective immobilization of tyrosinase via enzyme catalytic polymerization of l-DOPA for highly sensitive phenol and atrazine sensing. Talanta 160:125–132. https://doi.org/10.1016/j.talanta.2016.07.003

18.	Yang Q, Qu Y, Bo Y, et al (2010) Biosensor for atrazin based on aligned carbon nanotubes modified with glucose oxidase. Microchim Acta 168:197–203. https://doi.org/10.1007/s00604-009-0272-x

19.	Kucherenko IS, Chen B, Johnson Z, et al (2021) Laser-induced graphene electrodes for electrochemical ion sensing, pesticide monitoring, and water splitting. Anal Bioanal Chem 413:6201–6212. https://doi.org/10.1007/s00216-021-03519-w

20.	Wu Y, Chen Y, Zhang S, et al (2019) Bifunctional S, N-Codoped carbon dots-based novel electrochemiluminescent bioassay for ultrasensitive detection of atrazine using activated mesoporous biocarbon as enzyme nanocarriers. Analytica Chimica Acta 1073:45–53. https://doi.org/10.1016/j.aca.2019.04.068

21.	Valera E, Ramón-Azcón J, Barranco A, et al (2010) Determination of atrazine residues in red wine samples. A conductimetric solution. Food Chemistry 122:888–894. https://doi.org/10.1016/j.foodchem.2010.03.030

22.	Giannetto M, Umiltà E, Careri M (2014) New competitive dendrimer-based and highly selective immunosensor for determination of atrazine in environmental, feed and food samples: The importance of antibody selectivity for discrimination among related triazinic metabolites. Analytica Chimica Acta 806:197–203. https://doi.org/10.1016/j.aca.2013.11.002

23.	Yang M, Zhao X, Zheng S, et al (2017) A new electrochemical platform for ultrasensitive detection of atrazine based on modified self-ordered Nb2O5 nanotube arrays. Journal of Electroanalytical Chemistry 791:17–22. https://doi.org/10.1016/j.jelechem.2017.03.009

24.	Liu X, Li W-J, Li L, et al (2014) A label-free electrochemical immunosensor based on gold nanoparticles for direct detection of atrazine. Sensors and Actuators B: Chemical 191:408–414. https://doi.org/10.1016/j.snb.2013.10.033

25.	Zumpano R, Manghisi M, Polli F, et al (2022) Label-free magnetic nanoparticles-based electrochemical immunosensor for atrazine detection. Anal Bioanal Chem 414:2055–2064. https://doi.org/10.1007/s00216-021-03838-y

26.	Chuc NV, Binh NH, Thanh CT, et al (2016) Electrochemical Immunosensor for Detection of Atrazine Based on Polyaniline/Graphene. Journal of Materials Science & Technology 32:539–544. https://doi.org/10.1016/j.jmst.2016.04.004

27.	Deep A, Saraf M, Neha, et al (2014) Styrene Sulphonic Acid Doped Polyaniline Based Immunosensor for Highly Sensitive Impedimetric Sensing of Atrazine. Electrochimica Acta 146:301–306. https://doi.org/10.1016/j.electacta.2014.09.048

28.	Norouzi P, Larijani B, Ganjali MR, Faridbod F (2012) Admittometric Electrochemical Determination of Atrazine by Nano-composite immune-biosensor using FFT- Square wave Voltammetry. International Journal of Electrochemical Science 7:10414–10426. https://doi.org/10.1016/S1452-3981(23)16873-8

29.	Bhardwaj SK, Bhardwaj N, Mohanta GC, et al (2015) Immunosensing of Atrazine with Antibody-Functionalized Cu-MOF Conducting Thin Films. ACS Appl Mater Interfaces 7:26124–26130. https://doi.org/10.1021/acsami.5b07692

30.	Tran HV, Yougnia R, Reisberg S, et al (2012) A label-free electrochemical immunosensor for direct, signal-on and sensitive pesticide detection. Biosensors and Bioelectronics 31:62–68. https://doi.org/10.1016/j.bios.2011.09.035

31.	González-Techera A, Zon MA, Molina PG, et al (2015) Development of a highly sensitive noncompetitive electrochemical immunosensor for the detection of atrazine by phage anti-immunocomplex assay. Biosensors and Bioelectronics 64:650–656. https://doi.org/10.1016/j.bios.2014.09.046

32.	Vaid K, Dhiman J, Kumar S, Kumar V (2023) Citrate and glutathione capped gold nanoparticles for electrochemical immunosensing of atrazine: Effect of conjugation chemistry. Environmental Research 217:114855. https://doi.org/10.1016/j.envres.2022.114855

33.	Dhamu VN, Poudyal DC, Telang CM, et al (2022) Electrochemically mediated multi-modal detection strategy-driven sensor platform to detect and quantify pesticides. Electrochemical Science Advances 2:e2100128. https://doi.org/10.1002/elsa.202100128

34.	Supraja P, Singh V, Vanjari SRK, Govind Singh S (2020) Electrospun CNT embedded ZnO nanofiber based biosensor for electrochemical detection of Atrazine: a step closure to single molecule detection. Microsyst Nanoeng 6:3. https://doi.org/10.1038/s41378-019-0115-9

35.	Wang Y, Sun H, Liu M, et al (2020) A novel self-powered aptasensor for environmental pollutants detection based on simple and efficient enzymatic biofuel cell. Sensors and Actuators B: Chemical 305:127468. https://doi.org/10.1016/j.snb.2019.127468

36.	Madianos L, Tsekenis G, Skotadis E, et al (2018) A highly sensitive impedimetric aptasensor for the selective detection of acetamiprid and atrazine based on microwires formed by platinum nanoparticles. Biosensors and Bioelectronics 101:268–274. https://doi.org/10.1016/j.bios.2017.10.034

37.	Du B, Lu H, Zhang Z, et al (2022) Self-powered aptasensor for picomole level pollutants based on a novel enzyme-free photofuel cell. Biosensors and Bioelectronics 216:114661. https://doi.org/10.1016/j.bios.2022.114661

38.	Zhu Q-Q, Li H-K, Sun X-L, et al (2021) Rational incorporation of covalent organic framework/carbon nanotube (COF/CNT) composites for electrochemical aptasensing of ultra-trace atrazine. J Mater Chem C 9:8043–8050. https://doi.org/10.1039/d1tc01506k

39.	Sun H, Feng Y, Zhang Z, et al (2023) Immobilization-Free Photoelectrochemical Aptasensor for Atrazine Based on Bifunctional Graphene Signal Amplification and a Controllable Sulfhydryl-Assembled BiOBr/Ag NP Microinterface. Anal Chem 95:15736–15744. https://doi.org/10.1021/acs.analchem.3c03219

40.	Fan L, Zhang C, Liang G, et al (2021) Highly sensitive photoelectrochemical aptasensor based on MoS2 quantum dots/TiO2 nanotubes for detection of atrazine. Sensors and Actuators B: Chemical 334:129652. https://doi.org/10.1016/j.snb.2021.129652

41.	Zhang Z, Ding X, Lu G, et al (2023) A highly sensitive and selective photoelectrochemical aptasensor for atrazine based on Au NPs/3DOM TiO2 photonic crystal electrode. Journal of Hazardous Materials 451:131132. https://doi.org/10.1016/j.jhazmat.2023.131132

42.	Chen Y, Zhang X, Huang W, et al (2025) Ultrasensitive detection of atrazine by Schottky junction photoelectrochemical aptamer sensor based on signal amplification by cascade catalysis of CRISPR/Cas12a and G-quadruplex/hemin DNAzyme. Microchim Acta 192:376. https://doi.org/10.1007/s00604-025-07231-9

43.	Fan L, Zhang C, Yan W, et al (2019) Design of a facile and label-free electrochemical aptasensor for detection of atrazine. Talanta 201:156–164. https://doi.org/10.1016/j.talanta.2019.03.114

44.	Zhang Z, Wang Y, Chen Q, et al (2024) Aptamer molecular gate functionalized mesoporous SiO2@MB controlled-release system for pollutant detection using Ti(Ⅲ) self-doped TiO2 NTs as active photoanode coupled with electrostatic modulation. Talanta 277:126409. https://doi.org/10.1016/j.talanta.2024.126409

45.	Han W, Xie L, Zhu L, et al (2024) RecJf exonuclease-catalyzed signal amplified aptasensor for sensitive detection of atrazine using Ni6MnO8@C/Au nanorods and COF@MOF nanohybrids. Sensors and Actuators B: Chemical 398:134769. https://doi.org/10.1016/j.snb.2023.134769

46.	Sun C, Liu M, Sun H, et al (2019) Immobilization-free photoelectrochemical aptasensor for environmental pollutants: Design, fabrication and mechanism. Biosensors and Bioelectronics 140:111352. https://doi.org/10.1016/j.bios.2019.111352

47.	Han W, Wei P, Xie L, et al (2025) Heteroatom modulation of nickel single-atom nanozymes for enhanced interfacial catalytic activity in sensitive electrochemical detection of herbicides. Chemical Engineering Journal 505:159557. https://doi.org/10.1016/j.cej.2025.159557

48.	Yan P, Jin Y, Xu L, et al (2022) Enhanced photoelectrochemical aptasensing triggered by nitrogen deficiency and cyano group simultaneously engineered 2D carbon nitride for sensitively monitoring atrazine. Biosensors and Bioelectronics 206:114144. https://doi.org/10.1016/j.bios.2022.114144

49.	Yue P, Ruiyi L, Xie W, et al (2025) Electrochemical biosensor based on Au5Ir@graphene quantum dot nanocomposite and DNA walker for detection of atrazine in environmental water with extremely high sensitivity and selectivity. Sensors and Actuators B: Chemical 423:136873. https://doi.org/10.1016/j.snb.2024.136873

50.	Tucci M, Bombelli P, Howe CJ, et al (2019) A Storable Mediatorless Electrochemical Biosensor for Herbicide Detection. Microorganisms 7:630. https://doi.org/10.3390/microorganisms7120630

51.	Antonacci A, Attaallah R, Arduini F, et al (2021) A dual electro-optical biosensor based on Chlamydomonas reinhardtii immobilised on paper-based nanomodified screen-printed electrodes for herbicide monitoring. J Nanobiotechnol 19:145. https://doi.org/10.1186/s12951-021-00887-4

52.	Shitanda I, Takada K, Sakai Y, Tatsuma T (2005) Compact amperometric algal biosensors for the evaluation of water toxicity. Analytica Chimica Acta 530:191–197. https://doi.org/10.1016/j.aca.2004.09.073

53.	Shitanda I, Takamatsu S, Watanabe K, Itagaki M (2009) Amperometric screen-printed algal biosensor with flow injection analysis system for detection of environmental toxic compounds. Electrochimica Acta 54:4933–4936. https://doi.org/10.1016/j.electacta.2009.04.005

54.	Zamaleeva AI, Sharipova IR, Shamagsumova RV, et al (2011) A whole-cell amperometric herbicide biosensor based on magnetically functionalised microalgae and screen-printed electrodes. Anal Methods 3:509–513. https://doi.org/10.1039/c0ay00627k

55.	Pandard P, Rawson DM (1993) An amperometric algal biosensor for herbicide detection employing a carbon cathode oxygen electrode. Environmental Toxicology and Water Quality 8:323–333. https://doi.org/10.1002/tox.2530080309

56.	Boron I, Juárez A, Battaglini F (2020) Portable Microalgal Biosensor for Herbicide Monitoring. ChemElectroChem 7:1623–1630. https://doi.org/10.1002/celc.202000210

57.	Husu I, Rodio G, Touloupakis E, et al (2013) Insights into photo-electrochemical sensing of herbicides driven by *Chlamydomonas reinhardtii* cells. Sensors and Actuators B: Chemical 185:321–330. https://doi.org/10.1016/j.snb.2013.05.013

58.	Tucci M, Grattieri M, Schievano A, et al (2019) Microbial amperometric biosensor for online herbicide detection: Photocurrent inhibition of *Anabaena variabilis*. Electrochimica Acta 302:102–108. https://doi.org/10.1016/j.electacta.2019.02.007

59.	Buyukharman M, Gover T, Gumus A, et al (2024) Design of a Novel Green Algae-Based Biological Photovoltaic Cell with High Photocurrent and a Photoelectrochemical Biosensing Approach Utilizing the BPV for Pesticide Analysis in Water. ChemistrySelect 9:e202304943. https://doi.org/10.1002/slct.202304943

60.	Buyukharman M, Mulazimoglu IE, Yildiz HB (2024) Construction of a Conductive Polymer/AuNP/Cyanobacteria-Based Biophotovoltaic Cell Harnessing Solar Energy to Generate Electricity via Photosynthesis and Its Usage as a Photoelectrochemical Pesticide Biosensor: Atrazine as a Case Study. ACS Omega 9:16249–16261. https://doi.org/10.1021/acsomega.3c10308

61.	Koblížek M, Malý J, Masojídek J, et al (2002) A biosensor for the detection of triazine and phenylurea herbicides designed using Photosystem II coupled to a screen‐printed electrode. Biotech &amp; Bioengineering 78:110–116. https://doi.org/10.1002/bit.10190

62.	Swainsbury DJK, Friebe VM, Frese RN, Jones MR (2014) Evaluation of a biohybrid photoelectrochemical cell employing the purple bacterial reaction centre as a biosensor for herbicides. Biosensors and Bioelectronics 58:172–178. https://doi.org/10.1016/j.bios.2014.02.050

63.	Buyukharman M, Vardar DO, Cam OG, Yildiz HB (2026) Environmentally benign biophotovoltaic cell using thylakoid membrane and graphene–cellulose nanocomposite for solar energy harvesting and photoelectrochemical monitoring of waterborne herbicides. Journal of Photochemistry and Photobiology A: Chemistry 472:116796. https://doi.org/10.1016/j.jphotochem.2025.116796

64.	Buyukharman M, Ashurov N, Atakhanov A, et al (2025) Electrospun graphene-cellulose acetate/*Paulschulzia pseudovolvox* (Algae) modified photoelectrodes generating electricity via harnessing solar energy: A novel biosensing design for photoelectrochemical pesticide detection via photosynthesis inhibition. Electrochimica Acta 525:146099. https://doi.org/10.1016/j.electacta.2025.146099

65.	Rasmussen M, Minteer SD (2013) Self-powered herbicide biosensor utilizing thylakoid membranes. Anal Methods 5:1140–1144. https://doi.org/10.1039/c3ay26488b

66.	Masojídek J, Souček P, Máchová J, et al (2011) Detection of photosynthetic herbicides: Algal growth inhibition test vs. electrochemical photosystem II biosensor. Ecotoxicology and Environmental Safety 74:117–122. https://doi.org/10.1016/j.ecoenv.2010.08.028

67.	Bhalla V, Zazubovich V (2011) Self-assembly and sensor response of photosynthetic reaction centers on screen-printed electrodes. Analytica Chimica Acta 707:184–190. https://doi.org/10.1016/j.aca.2011.09.020

68.	Antonacci A, Frisulli V, Carvalho LB, et al (2023) An All-Green Photo-Electrochemical Biosensor Using Microalgae Immobilized on Eco-Designed Lignin-Based Screen-Printed Electrodes to Detect Sustainable Nanoherbicides. International Journal of Molecular Sciences 24:10088. https://doi.org/10.3390/ijms241210088

69.	Attaallah R, Antonacci A, Mazzaracchio V, et al (2020) Carbon black nanoparticles to sense algae oxygen evolution for herbicides detection: Atrazine as a case study. Biosensors and Bioelectronics 159:112203. https://doi.org/10.1016/j.bios.2020.112203
